# Screening the receptors for *Mycoplasma penetrans* P35 lipoprotein and characterization of its functional binding domains

**DOI:** 10.3389/fcimb.2025.1525789

**Published:** 2025-03-17

**Authors:** Xia Li, Xiaoliu Wang, Youyuan Ye, Zhuo Zeng, Li Chen, Kailan Peng, Hua Xiao, Siqi Gao, Haodang Luo, Yanhua Zeng

**Affiliations:** ^1^ Institute of Pathogenic Biology, Basic Medical School, Hengyang Medical College, University of South China; Hunan Provincial Key Laboratory for Special Pathogens Prevention and Control, Hengyang, Hunan, China; ^2^ Department of Dermatology and Venereology, The First Affiliated Hospital, Hengyang Medical College, University of South China, Hengyang, Hunan, China

**Keywords:** *Mycoplasma penetrans*, P35 lipoprotein, ACTG1, receptor, binding functional domains

## Abstract

*Mycoplasma penetrans*, a prokaryotic microorganism initially isolated from the urine of a patient infected with *human immunodeficiency virus* (HIV), possesses a distinctive elongated flask-like shape and a tip-like structure. This unique morphology has been shown to facilitate its ability to invade cells both *in vitro* and *in vivo*. The adhesion of *M. penetrans* to host cells relies on lipid-associated membrane proteins (LAMPs), especially P35 lipoprotein, which is exposed on the mycoplasmal surface. In this study, modified Virus Overlay Protein Binding Assay (VOPBA) was employed to identify P35-interacting proteins from membrane protein extracts of SV40-immortalized human uroepithelial (SV-HUC-1) cells. Through recombinant protein binding assays, siRNA-mediated knockdown, ELISA, Far-Western blot, and inhibition experiments, the binding mechanisms and functional domains were further elucidated. Results demonstrated that the P35 lipoprotein interacts with γ-actin (ACTG1). Recombinant P35 specifically bound to both recombinant and endogenous ACTG1 on the host cell membrane. ACTG1 partially inhibited the adhesion of P35 and *M. penetrans* to host cells. In SV-HUC-1 cells transfected with ACTG1-siRNA, adhesion of P35 and *M. penetrans* was significantly reduced. Further studies identified the functional domains responsible for binding between P35 and ACTG1 at amino acid residues 35-42 and 179-186. These findings suggest that ACTG1 on the host cell membrane may act as a receptor for the P35 lipoprotein, facilitating the adhesion of *M. penetrans* to host cells. The identified critical binding regions of P35 represent potential targets for therapeutic interventions against *M. penetrans* infections.

## Introduction

*Mycoplasma* is the smallest self-replicating prokaryotic microorganism, which evolved from the genome reduction of gram-positive bacteria. The loss of genes related to metabolic activity and biosynthesis has rendered all known *Mycoplasma* species obligate parasites, conferring strict host and tissue specificity (Rottem, 2003; Schwab et al., 2023). Lacking a cell wall, *Mycoplasma* adapts to various host environments primarily through its cell membrane (Melgaço et al., 2018). The mycoplasmal membrane consists of a three-layered structure that directly interacts with the external environment and is characterized by a single, restricted plasma membrane enriched with lipoproteins, known as lipid-associated membrane proteins (LAMPs). These LAMPs play critical roles in *Mycoplasma* adhesion, colonization, and invasion of host cells (Huang et al., 2018; He et al., 2014). Mycoplasmal lipoproteins are potent immunogens, eliciting strong and specific humoral immune responses during natural infections (Neyrolles et al., 1999a; Chen et al., 2021). Some lipoproteins undergo antigenic variation, facilitating immune evasion and contributing to chronic infections (Yueyue et al., 2022; Ferraz et al., 2007). Additionally, surface-exposed lipoproteins are primary targets for the host immune system (Lo et al., 1992). Therefore, understanding the interactions between mycoplasmal lipoproteins and host cell receptors is essential for elucidating the molecular mechanisms underlying *Mycoplasma* pathogenesis.

*Mycoplasma penetrans*, first isolated in 1991 from the urine of HIV-infected individuals, is a human pathogenic mycoplasma (Wang et al., 1992). Seroepidemiological studies have identified *M. penetrans* as a cofactor in the progression of HIV-infected people to AIDS, earning it the designation of AIDS-associated *Mycoplasma* (Gardette et al., 2023; Srinivasan et al., 2021). However, *M. penetrans* has also been isolated from blood and respiratory tract cultures of non-HIV-infected individuals with bacteremia and primary antiphospholipid syndrome (Neyrolles et al., 1999a; Srinivasan et al., 2021; Preiswerk et al., 2020), which indicates that *M. penetrans* can be pathogenic even in the absence of HIV. Recent studies have further associated *M. penetrans* with nongonococcal urethritis (Srinivasan et al., 2021; Schwab et al., 2023). However, the isolation of *M. penetrans* from clinical samples is challenging, resulting in its clinical diagnosis being almost entirely dependent on serological assays using an antigenic formulation derived from the Triton X-114 extract of the *Mycoplasma* (Wang et al., 1992). The extract comprises two primary LAMPs, P35 (35 kDa) and P38 (38 kDa) (Neyrolles et al., 1999a; Lo et al., 2005). The P35 lipoprotein is an immunodominant antigen exposed on the *Mycoplasma* surface and is implicated in mediating cell adhesion and invasion (Girón et al., 1996; Lo et al., 1993). In contrast, the P38 lipoprotein is not surface-exposed and can cross-react with sera containing antibodies against *M. pneumoniae* or *Ureaplasma urealyticum* (Neyrolles et al., 1999a). Consequently, P35 is typically used as the target antigen for the specific serological diagnosis of *M. penetrans* (Röske et al., 2001). Moreover, P35 is the first antigen recognized by the host immune system during humoral immune responses against *M. penetrans* (Lo et al., 2005; Neyrolles et al., 1999b). Despite its established importance in *M. penetrans* infection, the mechanisms by which P35 interacts with host cell receptors remain inadequately understood.

In this study, we focused on the receptors that mediate *M. penetrans* to adhere and even invade SV-HUC-1 cells. The results showed that γ-actin (ACTG1) was the main adhesion receptor of *M. penetrans* P35 lipoprotein on the SV-HUC-1 cell membrane. The amino acids at positions 35-42 and 179-186 of P35 were the functional binding domains for the interaction between P35 and ACTG1. The findings of this study contribute to a better understanding of the adhesion and pathogenic mechanisms of *M. penetrans* in host cells and lay the groundwork for the effective prevention and treatment of *M. penetrans* infections by targeting ACTG1 or its binding domains.

## Materials and methods

### Cell lines, bacterial strains and cultivation

SV-HUC-1 cells (ATCC, CRL-9520) were obtained from the Cell Bank of Chinese Academy of Sciences (Shanghai, China) and cultured in F-12k medium (Gibco, USA) supplemented with 12.5% fetal bovine serum (Gibco, USA). *M. penetrans* GTU-54-6Al was a kind gift from Professor Jiwen Zhao of Southeast University and has been preserved by the Institute of Pathogenic Biology of Hengyang Medical School University of South China. *M. penetrans* was cultured using SP-4 medium at 37°C.

### Expression of recombinant P35 protein and the synthesis of polypeptides

The recombinant protein coded by the DNA sequence of the *M. penetrans* P35 lipoprotein (protein ID: AAC16392.1) was expressed and purified as follows. The full-length DNA segment of *M. penetrans* P35 lipoprotein gene was codon-optimized and inserted into the pET-30a(+) vector. The recombinant prokaryotic expression vector pET-30a(+)/P35 was transformed into *E. coli* Rosetta™2 (DE3), which was induced by Isopropyl β-D-1-thiogalactopyranoside (IPTG; Solarbio, 0.5 mM) to express the recombinant hexahistidine-tagged P35 (rP35). After centrifugation at 10 000 rpm for 15 min, the induced expression solution was precipitated with PBS containing 0.1 mM PMSF and lysed by ultrasound at low temperature until the solution became clear. Then the bacteria solution was centrifuged at 12 000 rpm for 15 min, the supernatant and precipitation were collected, and the presence of protein was analyzed by SDS-PAGE. The supernatant was loaded into a column containing Ni-NTA immunoaffinity chromatography and incubated for 6 hours by shock on ice. The effluent was collected and eluted with a linear gradient of 20 to 150mM imidazole. The effluents were analyzed by SDS-PAGE electrophoresis. The purified rP35 was washed with PBS and concentrated on a regenerated cellulose membrane (Millipore, USA) with a molecular weight cutoff of 10 kDa.

Based on secondary structure and hydrophilicity analyses of the P35 lipoprotein, as well as epitope mapping and recognition rates by human serum reported in literatures (Neyrolles et al., 1999b; Sasaki et al., 2002), five potential binding-related peptides were synthesized. Each truncated P35 peptide was tagged with a Flag epitope (DYKDDDDK) using a flexible linker (GGGGSGGGGS). A control peptide containing only the Flag tag and linker sequences (GGGGSGGGGSDYKDDDDK) was also synthesized (**Table 1**). Peptide synthesis, identification, and purification were performed by Nanjing GenScript Co., Ltd. Rabbit-derived anti-Flag antibody was purchased from Biopm (PMK101M, China).

**Table 1:** Peptides used in this study.

Peptide	Sequence	Amino acids
rP35-1	SENNGNGNGGGGSGGGGSDYKDDDDK	35-42
rP35-2	ANPENYFTGGGGSGGGGSDYKDDDDK	95-102
rP35-3	FTGEAYSVGGGGSGGGGSDYKDDDDK	127-134
rP35-4	PNLKLNNGGGGGSGGGGSDYKDDDDK	179-186
rP35-5	DSTNNNKYGGGGSGGGGSDYKDDDDK	323-330
Negative peptide	GGGGSGGGGSDYKDDDDK	

Flexible Linker (GGGGSGGGGS), Flag tag (DYKDDDDK).

### Preparation of polyclonal antibody

Three 8-week-old female New Zealand rabbits were purchased from Hunan Taiping Biotechnology Co., LTD. The New Zealand rabbits were immunized using the purified rP35 (150μg) mixed with Freund’s adjuvant. After four immunizations, rabbit serum was collected and antibody titer was determined. The collected rabbit serum was centrifuged 12,000 rpm for 30 minutes, and the supernatants were collected. The supernatants were roughly purified by continuous ammonium sulfate precipitation, and then further purified by CNBr-activated Sepharose 4B (GE Healthcare, Sweden) as described by our group (Peng et al., 2023; Zeng et al., 2012). All antibodies were diluted with TBST (0.05% Tween 20 in Tris-buffered saline) containing 5% skim milk.

### Transfection of small interfering RNA

When SV-HUC-1 cells were cultured to approximately 10^5^-10^6^ cells, the nutrient medium was changed into fresh serum-free F-12K medium and continued to culture overnight. Then, the ACTG1-siRNA (20 mM, Origin, SR300049, USA) was transfected into the cells using Lipofectamine 3000. After 48 hours of transfection, the medium was supplemented with F-12K containing 10% fetal bovine serum for subsequent experiments.

### Western blot

Protein samples were mixed with loading buffer and boiled for 10 minutes. Proteins were then separated by 10–15% gradient acrylamide gel electrophoresis and transferred to polyvinylidene fluoride (PVDF) membranes (Millipore, R0BB23469, USA). The membranes were blocked for 2 hours at room temperature with TBST containing 5% skim milk, followed by three washes. Membranes were incubated overnight at 4°C with the appropriate primary antibodies. Mouse IgG antibody or rabbit IgG antibody served as negative controls. After washing four times, membranes were incubated with horseradish peroxidase (HRP)-conjugated goat anti-rabbit or anti-mouse IgG secondary antibodies (1:5,000, Proteintech, SA00001-2 or SA00001-1, USA) at 37°C for 1 hour. Following final washes and drying, PVDF membranes were subjected to chemiluminescent detection using BeyoECL Star (Beyotime, China).

### Modified VOPBA and HPLC-MS

The SV-HUC-1 membrane proteins were extracted as described in our previous study (Deng et al., 2018). The protein samples were boiled for 5 minutes, electrophoreted with 12.5% acrylamide gel, and then stained with Coomassie blue. Molecular masses were estimated based on the Protein Ladder (Thermo, 26616, USA). To detect P35 binding proteins on the SV-HUC-1 membrane, the membrane proteins were transferred to PVDF membranes, which were then blocked in TBST containing 5% skim milk at room temperature for 2 hours, and incubated with rP35 (0.5 mg/mL) at 4°C overnight. BSA incubation was served as a negative control. After washing, the membranes were incubated with purified rabbit anti-rP35 antibody (1:50) at 37°C for 2 hours. Incubation of secondary antibody and visualization and recording of protein bands were performed as described in western blot. The protein band intensities were quantified by ImageJ.

After the molecular weight of the target bands were determined according to the results of Coomassie blue, an expected band (kDa) in the improved VOPBA experiment were cut and analyzed using high performance liquid chromatograph-mass spectrometry (HPLC-MS) by Guangzhou Fitgene Biotechnology Co. Ltd.

### Distribution of target proteins on the cell membrane

SV-HUC-1 cells were inoculated into the 24-wells plate (Thermo Fisher, USA) and until reaching a density of 2 × 10^5^ cells at 37°C. Cells were washed twice using PBS and then fixed with 4% paraformaldehyde at 4°C for 30 minutes. After four washes, cells were blocked with F-12K complete medium as a blocker at room temperature for 1 hour. Following additional washes, cells were incubated overnight at 4°C with either rabbit anti-ACTG1 antibody (1:2,000, Antibody System, PHF60501, France) or rabbit anti-KRT8 antibody (1:2,000, Antibody System, PHC17901, France). After PBS washes, cells were incubated with anti-rabbit Alexa-488 secondary antibody (1:200, Jackson, 111-545-003, USA) for 1 hour at 37°C. Nuclei were stained with DAPI (4′,6′-diamidino-2-phenylindole, Beyotime, China). Images were captured using a TE2000-S inverted microscope (Nikon, Japan) at 40× magnification. Fluorescence images were captured sequentially to prevent crosstalk between fluorophores.

### Far-western blot

As previously described (Peng et al., 2023), far-western blot was used to identify whether full-length rP35 specifically interacts with recombinant target proteins (ACTG1, AtaGenix, ATAP01698, France; KRT8, Antibody System, YHC17901, France) and membrane proteins from SV-HUC-1 cells. Briefly, rP35 samples were boiled for 10 minutes, and then transferred to PVDF membranes. After washing and blocking, membranes were incubated overnight at 4°C with either membrane proteins (500 μg/mL) or the corresponding recombinant target proteins (ACTG1 or KRT8). Following PBS washes, membranes were incubated with rabbit anti-ACTG1 antibody (1:2,000) or rabbit anti-KRT8 antibody (1:2,000) for 2 hours at 37°C, followed by incubation with HRP-conjugated goat anti-rabbit secondary antibody (1:5,000) for 1 hour at 37°C. Protein bands were visualized and recorded as described in the Western Blot section.

Far-western blot was also performed to confirm the interaction between synthesized P35 peptides and ACTG1. Recombinant ACTG1 protein was transferred to the PVDF membranes as described above. After washing and blocking, PVDF membranes were incubated overnight at 4°C with synthesized P35 peptides. Full-length rP35 and a negative peptide served as positive and negative controls, respectively. Following PBS washes, membranes were incubated with rabbit anti-Flag antibody (1:2,000) for 2 hours at 37°C. Secondary antibody incubation, visualization, and recording of protein bands were performed as described in the Western Blot section.

### Indirect enzyme-linked immunosorbent assay

Interactions between full-length rP35 and ACTG1 or KRT8 were assayed using an indirect ELISA. Full-length rP35 (10 μg/well) was suspended in 0.1 M sodium carbonate solution (15 mM Na_2_CO3, 35 mM NaHCO3, pH 9.6) and immobilized onto a 96-well polystyrene plate (Corning, USA) overnight at 4°C. After washing with PBST (0.05% Tween 20 in PBS) three times, plates were blocked with 5% skimmed milk in TBST for 2 hours at 37°C. After three additional washes with PBST, wells were incubated with either ACTG1 (0.2 mg/mL) or KRT8 (0.2 mg/mL, Antibody System, China) for 2 hours at 37°C. Wells incubated with BSA were served as negative control, and those with rabbit anti-rP35 antibodies (1:200) served as positive controls. Following washes, wells were incubated with rabbit anti-ACTG1 antibodies (1:2,000, Antibody System) or rabbit anti-KRT8 antibodies (1:2,000, Antibody System) for 2 hours at 37°C. After five washes with PBST, wells were incubated with HRP-conjugated goat anti-rabbit IgG antibody (1:5,000) for color development. Absorbance at 450 nm (A_450_) was measured using a microplate meter (Tecan, Infinite F50, Switzerland).

ELISA was also utilized to detect the binding of truncated P35 polypeptides to immobilized ACTG1. ACTG1 (10 μg/well; AtaGenix) was immobilized on the plate and incubated with synthesized P35 polypeptides (100 μg/mL) for 2 hours at 37°C. Wells incubated with the control peptide served as negative control, and those with full-length rP35 served as positive control. Rabbit anti-Flag antibody (1:2,000, Biopm, PMK101M, China) or purified rabbit anti-P35 antibody was subsequently added, followed by incubation with HRP-conjugated goat anti-rabbit antibody (1:5,000). Absorbance at 450 nm (A450) was measured.

### Co-localization assay

To evaluate the co-localization of rP35 or *M. penetrans* with ACTG1, SV-HUC-1 cells were pre-incubated with rP35 (50 μg/mL) or *M. penetrans* (1×10^7^ CCU/mL) for 2 hours at 37°C. After washing, cells were incubated overnight at 4°C with a combination of mouse monoclonal anti-ACTG1 antibody and rabbit anti-rP35 antibodies (1:200) or rabbit anti-Mpe serum (1:10,000) in F-12K medium. Following PBS washes, cells were stained with a combination of anti-rabbit Alexa-488 (1:200) and Cy3 anti-mouse antibody (1:200, Proteintech, China) for 1 hour at 37°C. Nuclei were stained with DAPI. Images were captured using a TE2000-S inverted microscope (Nikon, Japan) at 100× magnification. Fluorescence images were captured sequentially to prevent crosstalk between fluorophores.

### Co- immunocoprecipitation assay

200 μL of membrane proteins extract were mixed with 100 μL rP35 (10 μg/mL) and shaken at 4°C overnight. Subsequently, 40 μL of 50% protein A/G-agarose beads (Santa Cruz, USA) working solution was added and incubated on ice for 2 hours to remove nonspecific proteins. Following centrifugation at 14,000 g, the supernatant was divided into five portions. One sample served as positive control, while the other four samples were incubated overnight at 4°C with rabbit anti-rP35 antibody (1:25), mouse anti-ACTG1 antibody (1:50, Abcam, ab52599, UK), mouse IgG (1:50; Servicebio, GB11739, China) and rabbit IgG (1:50, Servicebio, GB111738, China), respectively. Each sample was then incubated with 10 μL of 50% protein A/G-agarose beads working solution on ice overnight to capture the antigen-antibody complexes. The precipitated protein complexs were washed three times and then re-suspended with 60 μL of PBS. Precipitated rP35-ACTG1 complexes were visualized and recorded as described in the Western Blot section.

### Adhesion detection assay

SV-HUC-1 cells, with and without ACTG1 knockdown via siRNA transfection, were pre-incubated with rP35 (50 μg/mL) or M. penetrans (1 × 10^7^ CCU/mL) for 2 hours at 37°C. Pre-incubated cells were fixed with 4% paraformaldehyde at 4°C for 30 minutes and washed with PBS. To detect adhering rP35 or *M. penetrans*, cells were incubated overnight at 4°C with either rabbit anti-rP35 antibodies (1:200) or rabbit anti-Mpe serum (1:10,000). Following PBS washes, cells were stained with Cy3 anti-rabbit secondary antibody (1:200) for 1 hour at 37°C and nuclei were stained with DAPI. Images were captured using a TE2000-S inverted microscope (Nikon, Japan) at 40× magnification.

### Adhesion inhibition assay

The *M. penetrans* suspension was incubated for 2 hours at 37°C with rabbit anti-P35 antibody or pre-immune serum, followed by overnight incubation with SV-HUC-1 cells. Cells were then fixed and blocked as described previously. After washing, cells were incubated overnight at 4°C with rabbit anti-Mpe serum (1:10,000). Secondary antibody incubation and image capture were performed as described in the Adhesion Detection Assay.

To further determine the role of ACTG1 in the adhesion of *M. penetrans* and rP35 to host cells, two sets of adhesion inhibition assays were implemented. In one group, *M. penetrans* or rP35 suspension was pre-incubated with ACTG1 for 2 hours at 37°C before incubation of the SV-HUC-1 cells. In another group, cells were pre-incubated with ACTG1 antibody (1:2000, Abcam) for 2 hours at 37°C before incubation with *M. penetrans* or rP35 suspension. The adhesion detection group served as the positive control. The addition of primary and secondary antibodies and the capture of images were described in adhesion detection experiments.

Adhesion inhibition assay also examined whether the pre-incubation of the synthesized P35 polypeptides affected the adhesion of rP35 to SV-HUC-1 cells. The cells were pre-incubated with synthesized P35 polypeptides for 2 hours at 37°C before incubation of the rP35 suspension. The adhesion detection group was the positive control, and the control peptide incubation group was the negative control. The cells were washed and then incubated with rabbit anti-rP35 antibody (1:200) overnight at 4°C. The addition of primary and secondary antibodies and the capture of images are described in adhesion detection experiments. The average integrated optical density of all groups of images was obtained using ImageJ, and then the staining intensity of rP35 or *M. penetrans* was quantitatively analyzed.

### Statistical analysis

All experiments were performed in triplicate, and data are presented as mean ± standard deviation (SD). Statistical significance was determined using Student’s t-test or one-way ANOVA, as appropriate. A p-value of <0.05 was considered statistically significant. Data analysis was conducted using GraphPad Prism.

## Results

### Successful expression and purification of rP35

The full length P35 protein of *M. penetrans* was successfully expressed, identified and purified to facilitate the screening of interacting proteins. As shown in **Supplementary Figure S1** there were expected bands in the supernatant and precipitation near the molecular weight 35 kDa, indicating that the rP35 protein was expressed successfully. The purified rP35 using Ni-NTA immunoaffinity chromatography was verified with anti-His6x antibody, and the results showed that polyclonal antibody was successfully generated against rP35.

### Preparation and purification of rP35 polyclonal antibody

Polyclonal rabbit antibodies against rP35 were generated by immunizing New Zealand rabbits with rP35 and subsequently purified using CNBr activated Sepharose 4B. As depicted in **Supplementary Figure S2** of the **Supplementary Material**, the titer of specific antibody reached 1:2,560,000 after four immunizations. SDS-PAGE analysis revealed the heavy and light chains of the anti-rP35 antibody at approximately 55 kDa and 25 kDa, respectively, confirming the successful purification of the antibody. Furthermore, Western blot validated the successful preparation of the rP35-specific antibody.

### P35 lipoprotein as an adhesion-associated protein of *M. penetrans*

**Figure 1:**
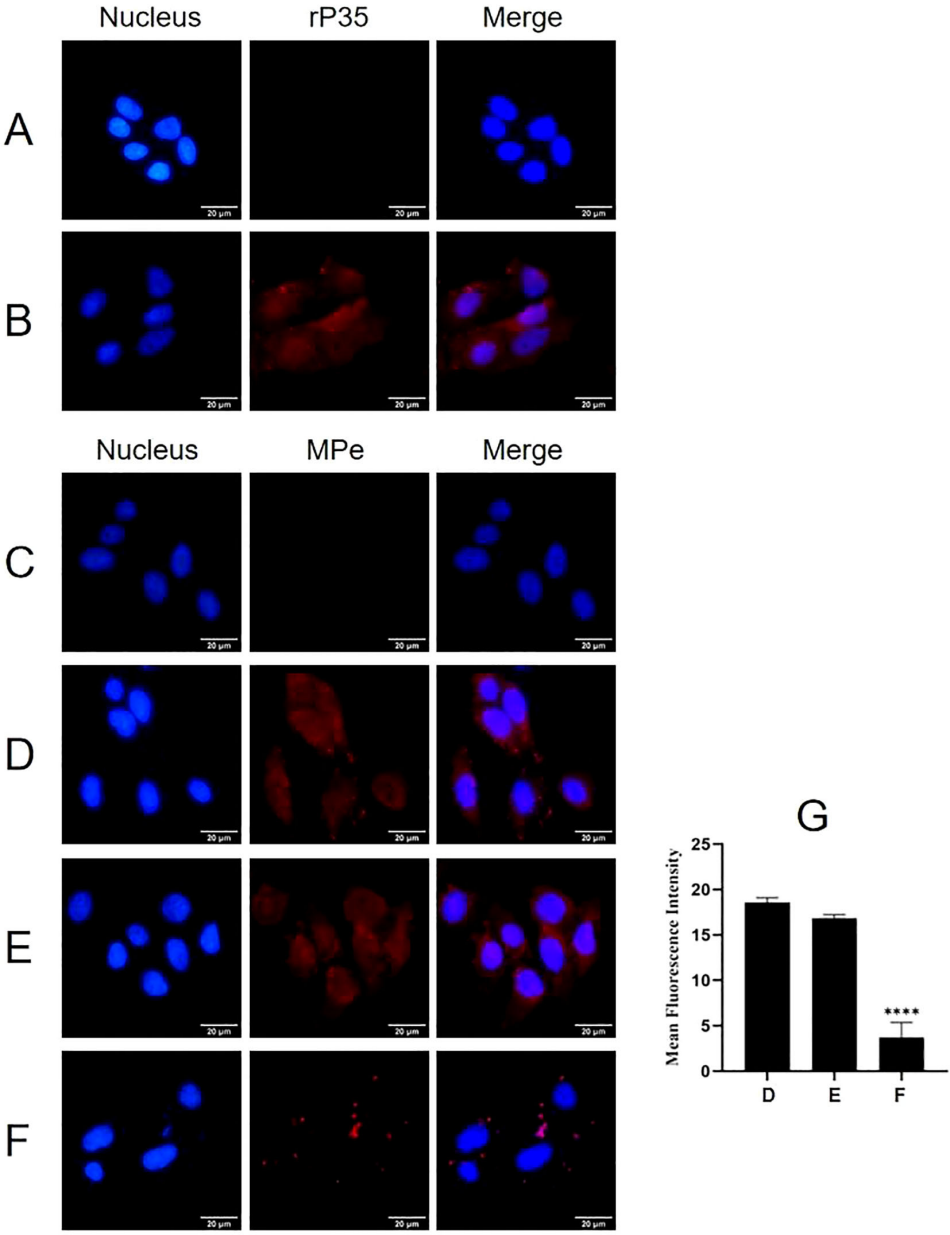
The adhesion and adhesion inhibition assay of rP35 and *M. penetrans* to SV-HUC-1 cells. **(A-D)** Anti-rP35 or anti-Mpe specific antibodies were used to detect rP35 or *M. penetrans* attached to SV-HUC-1 cells. Cells were incubated with rP35 **(B)** or *M. penetrans* **(D)**. Cells did not incubate with rP35 **(A)** or *M. penetrans* **(C)** were served as blank controls. After being fixed and blocked, the cells were incubated with rabbit anti-rP35 antibody or anti-Mpe serum and then stained with Cy3-conjugated secondary antibody (Red). **(E, F)** *M. penetrans* was pre-incubated with rabbit serum before immunization **(E)** or anti-rP35 antibody **(F)** before infection of the SV-HUC-1 cells. The cells were incubated with rabbit anti-*M. penetrans* antibody and then stained with Cy3-conjugated secondary antibody (Red). The nucleus was stained with DAPI (blue). The images were captured by fluorescence microscopy with 40× and represent multiple areas on multiple slides. **(G)** Quantitation of the staining intensities of *M. penetrans* from fluorescence microscopy. Data points represent the average of three times experiments; ****, statistical significance, *p* value < 0.0001, compared with the same concentration of *M. penetrans* adhesion group.

Indirect immunofluorescence was employed to determine whether P35 mediates the adhesion of *M. penetrans* to SV-HUC-1 cells as an adhesion-associated protein. As illustrated in **Figures 1B, D**, significant red fluorescence was observed on the surface of SV-HUC-1 cells incubated with rP35 or *M. penetrans*. In contrast, no obvious red fluorescence was detected in the blank control group (**Figures 1A, C**). These observations indicate that both rP35 and *M. penetrans* are capable of adhering to the surface of SV-HUC-1 cell.

Adhesion inhibition assays further demonstrated that compared with the *M. penetrans* adhesion group (**Figure 1D**), the red fluorescence on the membrane surface of SV-HUC-1 cells pretreated with pre-immune serum did not change significantly (**Figure 1E**). Conversely, the red fluorescence on the membrane surface of cells pretreated with the anti-rP35 antibody was markedly reduced (**Figure 1F**). Quantitative analysis of the average integrated optical density (**Figure 1G**) confirmed that the rP35 antibody effectively inhibited the adhesion of *M. penetrans* to SV-HUC-1 cells. Collectively, these findings establish P35 as an adhesion-associated protein facilitating the interaction between *M. penetrans* and SV-HUC-1 cells.

### Identification of membrane proteins binding to rP35

**Figure 2:**
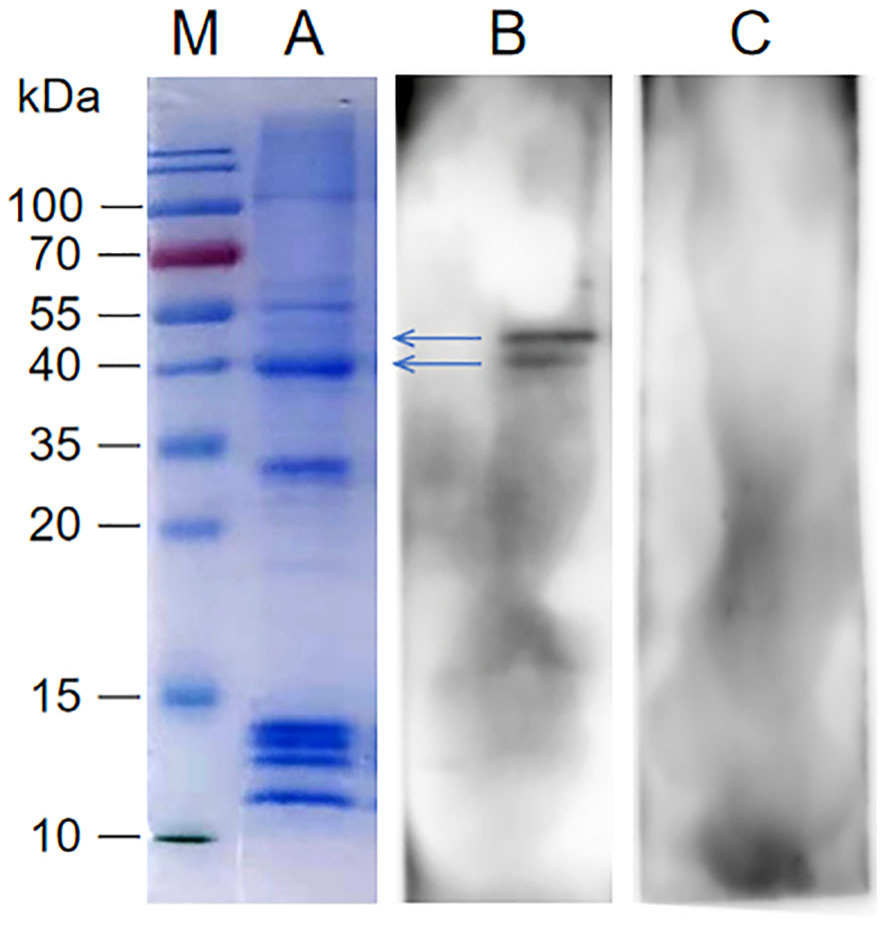
Screening of SV-HUC-1 membrane proteins specifically bind to rP35. Lane: M. Protein marker; **(A)** SDS-PAGE analysis of SV-HUC-1 membrane proteins; **(B, C)** SV-HUC-1 membrane proteins were isolated by SDS-PAGE and then were transferred to PVDF membranes, which was blocked and pre-incubated with rP35 (Lane B) or BSA (Lane C). After washing, anti-rP35 antibody was used to detect P35-binding membrane proteins.

SDS-PAGE analysis and modified VOPBA conducted to screen the interacting proteins of rP35. As shown in the **Figure 2A**, the molecular weights of SV-HUC-1 membrane proteins were mainly in the range of 10 kDa~100 kDa. The results of the modified VOPBA manifested that PVDF membrane incubated with rP35 demonstrated two distinct bands in the molecular weight range of 40 kDa to ~55 kDa (**Figure 2B**), while no obvious band appeared in the control group (**Figure 2C**). These results indicated that SV-HUC-1 membrane proteins in the range of 40 kDa to ~55 kDa might be the target proteins specifically binding to rP35.

To identify proteins that specifically bound to rP35, strips near 40 kDa~55 kDa were cut for HPLC-MS analysis. After protein matching and comparison search through NCBI database, it was found that ACTG1 (Actin Gamma 1) near 40 kDa had the highest score (**Supplementary Table 1**), and KRT8 (Keratin, type II cytoskeletal 8) near 50 kDa had the highest score (**Supplementary Table 1**). Therefore, ACTG1 and KRT8 are likely membrane proteins that specifically interact with rP35.

### Localization of ACTG1 and KRT8 in SV-HUC-1 cells

**Figure 3:**
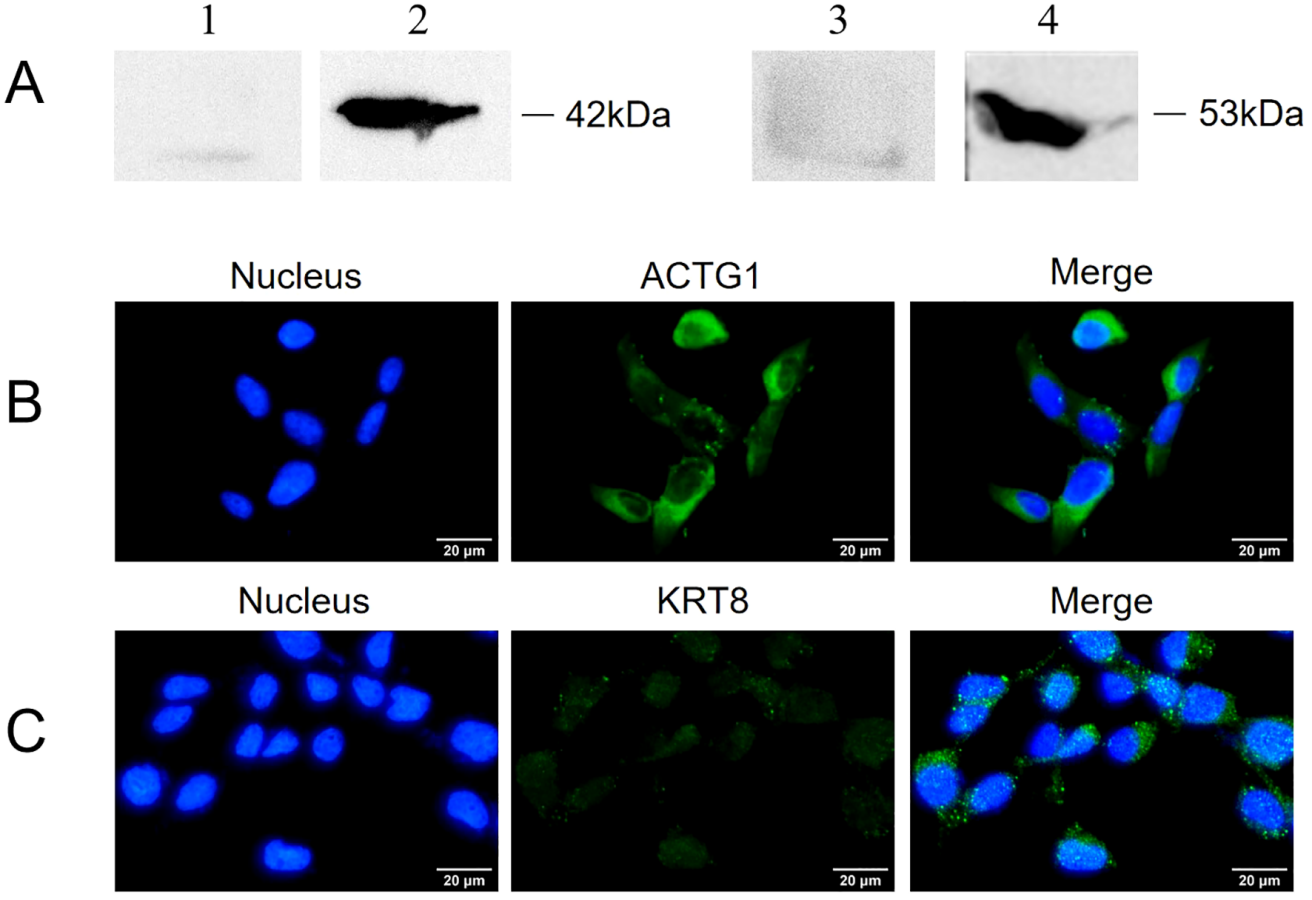
Expression of ACTG1 and KRT8 in SV-HUC-1 cells. **(A)** ACTG1 and KRT8 in membrane proteins of SV-HUC-1 cells were detected by Western blot with different antibodies. Lane: 1. Control, Rabbit IgG; 2. anti-ACTG1 antibodies; 3. Control, Rabbit IgG; 4. anti-KRT8 antibody. **(B, C)** Immunofluorescence images of SV-HUC-1 cells. The cell nucleus was stained with DAPI (blue), and the ACTG1 or KRT8 on the cells was stained with Alexa-488-labeled secondary antibody (green). The images were captured at 40× magnification and are representative of various regions across multiple slides.

The localization of ACTG1 and KRT8 within SV-HUC-1 cells was assessed using Western blot and indirect immunofluorescence. As depicted in **Figure 3A**, both ACTG1 and KRT8 were present in the membrane components of SV-HUC-1 cells. Indirect immunofluorescence assays further revealed that green fluorescence representing ACTG1 (**Figure 3B**) and KRT8 (**Figure 3C**) was localized in the cytoplasm and on the cell membrane. These results indicate that ACTG1 and KRT8 are predominantly distributed in the cytoplasm and membrane of SV-HUC-1 cells.

### Interaction between ACTG1 and rP35

**Figure 4:**
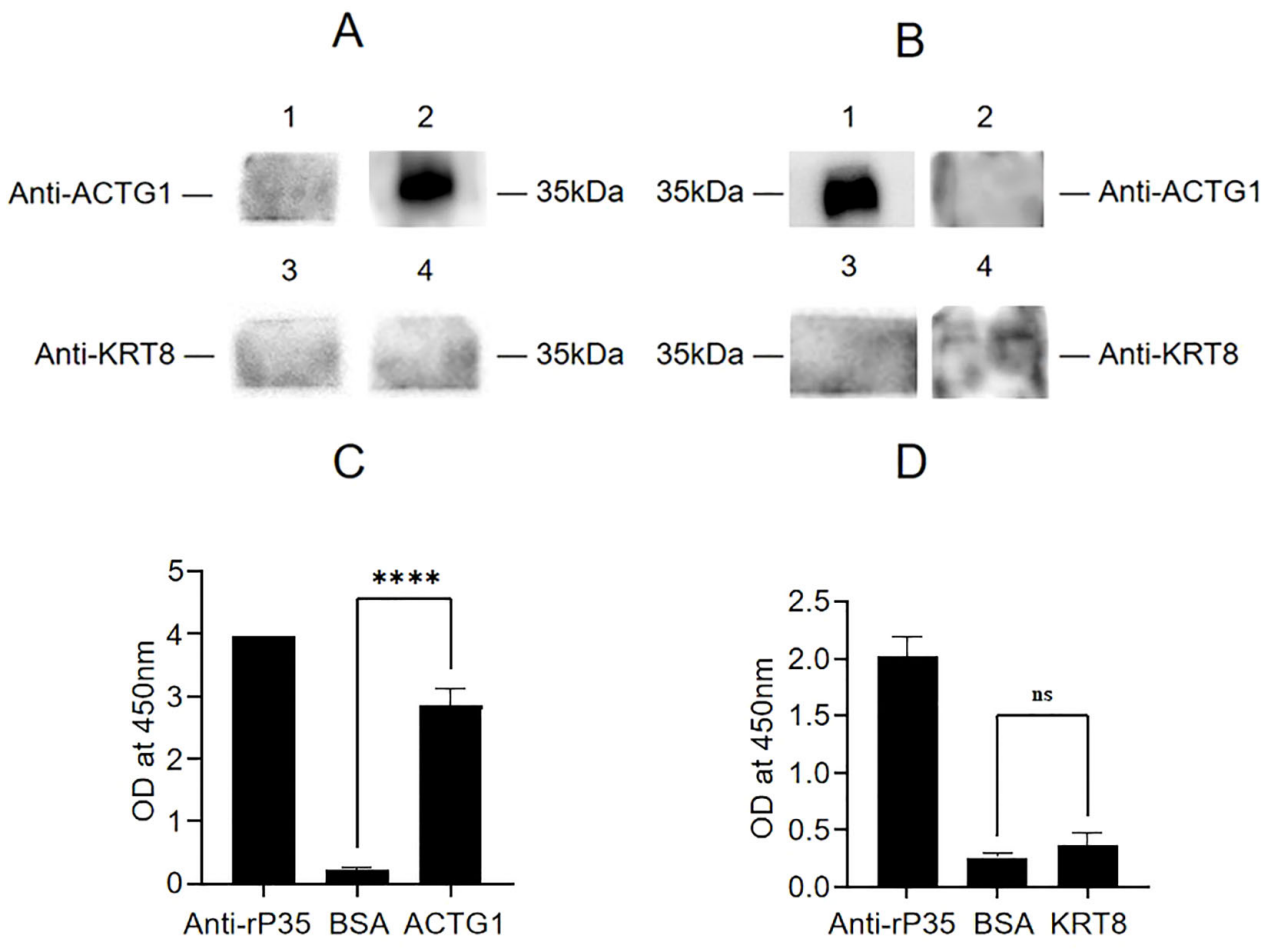
Binding analysis between ACTG1 or KRT8 protein and rP35. **(A)** Interaction of rP35 with ACTG1 or KRT8 was visualized by far-western blot. rP35 was separated by 12.5% SDS-PAGE and transferred to PVDF membrane, which was pre-incubated with ACTG1, KRT8 or BSA and probed with anti-ACTG1 antibody or anti-KRT8 antibody followed by HRP-conjugated goat anti-rabbit IgG antibody. Lane 1, PVDF was incubated with BSA and anti-ACTG1 antibody; Lane 2, PVDF was incubated with ACTG1 and anti-ACTG1 antibody; Lane 3, PVDF was incubated with BSA and anti-KRT8 antibody; Lane 4, PVDF was incubated with KRT8 and anti-KRT8 antibody. **(B)** Interaction between ACTG1 or KRT8 in SV-HUC-1 cells membrane and rP35 was visualized by far-western blot. Lane 1, PVDF was incubated with SV-HUC-1 cell membrane proteins and anti-ACTG1 antibody; Lane 2, PVDF was incubated with BSA and anti-ACTG1 antibody; Lane 3, PVDF was incubated with membrane proteins and anti-KRT8 antibody; Lane 4, PVDF was incubated with BSA and anti-KRT8 antibody. **(C)** Binding of ACTG1 to immobilized rP35 was detected using ELISA. **(D)** Binding of KRT8 to immobilized rP35 was tested using ELISA. ****P < 0.0001.

To determine whether KRT8 and ACTG1 interact with rP35, four independent approaches were used. First, the results of far-western blot were shown in **Figure 4A**, there were bands at 35 kDa for the recombinant ACTG1 protein incubated group, while there was no band in the control group, indicating that ACTG1 can directly bind to rP35. However, no obvious band appeared in the KRT8 protein incubated group, indicating that KRT8 protein could not directly bind to rP35. Additionally, we further verified whether rP35 could bind to ACTG1 and KRT8 on SV-HUC-1 cell membranes using far-western blot. As shown in **Figure 4B**, an obvious band at 35 kDa was observed for the ACTG1 antibody group, while no band was appeared for both the KRT8 antibody group and the control group. These results substantiated that rP35 could interact with ACTG1 on SV-HUC-1 cell membrane, but not with KRT8.

Second, the interaction of ACTG1 or KRT8 with rP35 were assayed by indirect ELISA. As shown in **Figures 4C, D**, the A_450_ values of rP35 and ACTG1 incubation group was significantly higher than that of the negative control group, while there was no significant difference between rP35-KRT8 incubation group and negative control group, indicating that rP35 specifically bound ACTG1 but did not interact with KRT8, which was consistent with the results of far-western blot.

**Figure 5:**
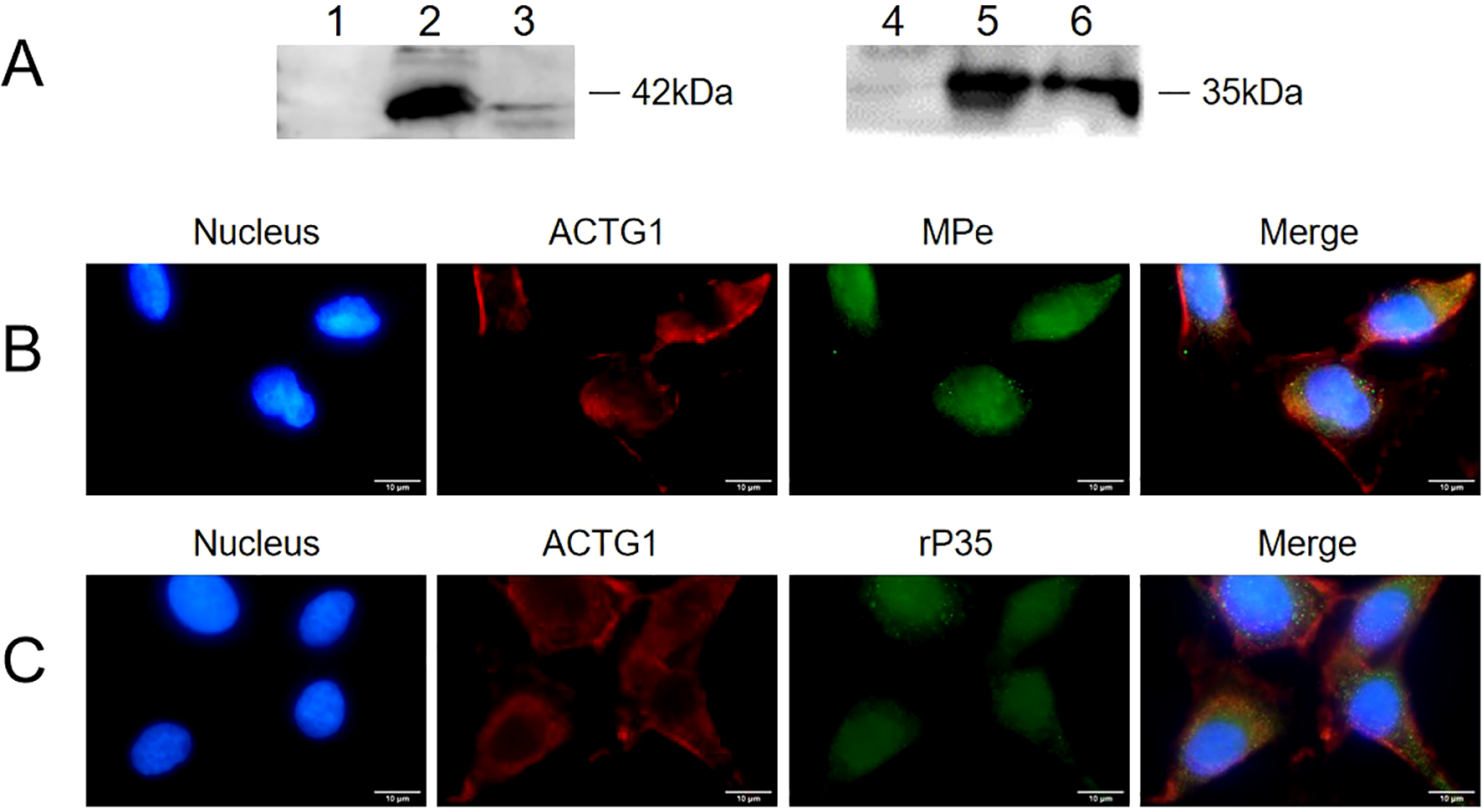
Interaction between ACTG1 and rP35 was detected using Co-immunoprecipitation and Co-localization analysis. **(A)** Co-immunoprecipitation was conducted using antibody targeting rP35 or ACTG1. The rP35 or ACTG1 binding was analyzed respectively using western blot. Lane: 1. Sample precipitated by IgG; 2. ACTG1-rP35 complex; 3. ACTG1-rP35 complex precipitated by anti-rP35 antibody, incubated with anti-ACTG1 antibody and visualized; 4. Sample precipitated by IgG; 5. ACTG1-rP35 complex; 6. ACTG1-rP35 complex precipitated by anti-ACTG1 antibody, incubated with anti-rP35 antibody and visualized. **(B)** Co-localization analysis between ACTG1 and *M. penetrans*. **(C)** Co-localization analysis between ACTG1 and rP35. ACTG1 was stained into red, while rP35 and *M. penetrans* were stained into green. All signals were merged (yellow) (×100, in oil). Representative images are shown.

Indirect immunofluorescence assay was performed to prove whether P35 can colocalize with ACTG1 in SV-HUC-1 cells. Representative fluorescence images in **Figures 5B, C** revealed that *M. penetrans* and the rP35 protein (green fluorescence) were detected on the cell membrane and co-localized with ACTG1 (red fluorescence), as shown in the merged images (yellow fluorescence).

**Figure 6:**
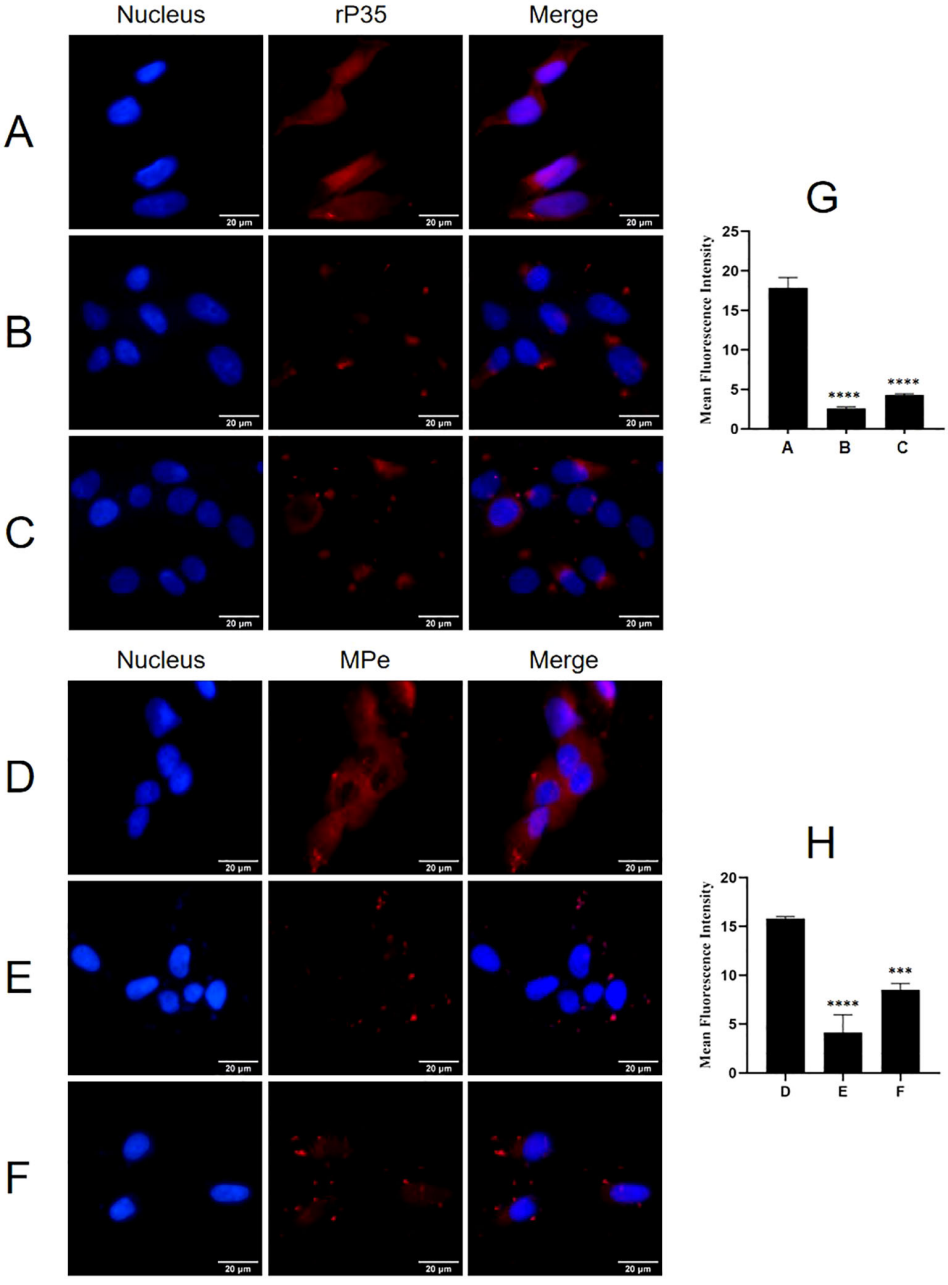
Effect of ACTG1 on the adhesion of rP35 and *M. penetrans* was determined using IFM. **(A)** Adhesion assay of rP35 on SV-HUC-1 cells was used as control group. **(B)** Adhesion inhibition assay of ACTG1-treated rP35 on SV-HUC-1 cells. **(C)** Adhesion assay of rP35 on SV-HUC-1 cells treated with anti-ACTG1 antibody. **(D)** Adhesion assay of *M. penetrans* on SV-HUC-1 cells was used as control group. **(E)** Adhesion inhibition assay of ACTG1-treated *M. penetrans* on SV-HUC-1 cells. **(F)** Adhesion assay of *M. penetrans* on SV-HUC-1 cells treated with anti-ACTG1 antibody. Nuclei were stained with DAPI (blue), and rP35 and *M. penetrans* were stained with Cy3 (red). The images were taken at a magnification of 40×and are representative of multiple areas on multiple slides. **(G, H)** The quantitation of the staining intensities of rP35 **(G)** and *M. penetrans* **(H)** from fluorescence microscopy. ***P < 0.001, ****P < 0.0001.

The interaction between ACTG1 and rP35 was further verified using immunoprecipitation. The results showed that both the anti-rP35 antibody and anti-ACTG1 antibody precipitated samples had obvious band at 42 kDa (**Figure 5A-3**) and 35 kDa (**Figure 5A-6**), respectively, while the control IgG precipitation group had no obvious band (**Figures 5A-1, 5A-4**). Collectively, these data corroborated that P35 could interact with ACTG1 on the cell membrane of SV-HUC-1 cells.

### Functional impact of ACTG1 on the adhesion of *M. penetrans* to rP35

**Figure 7:**
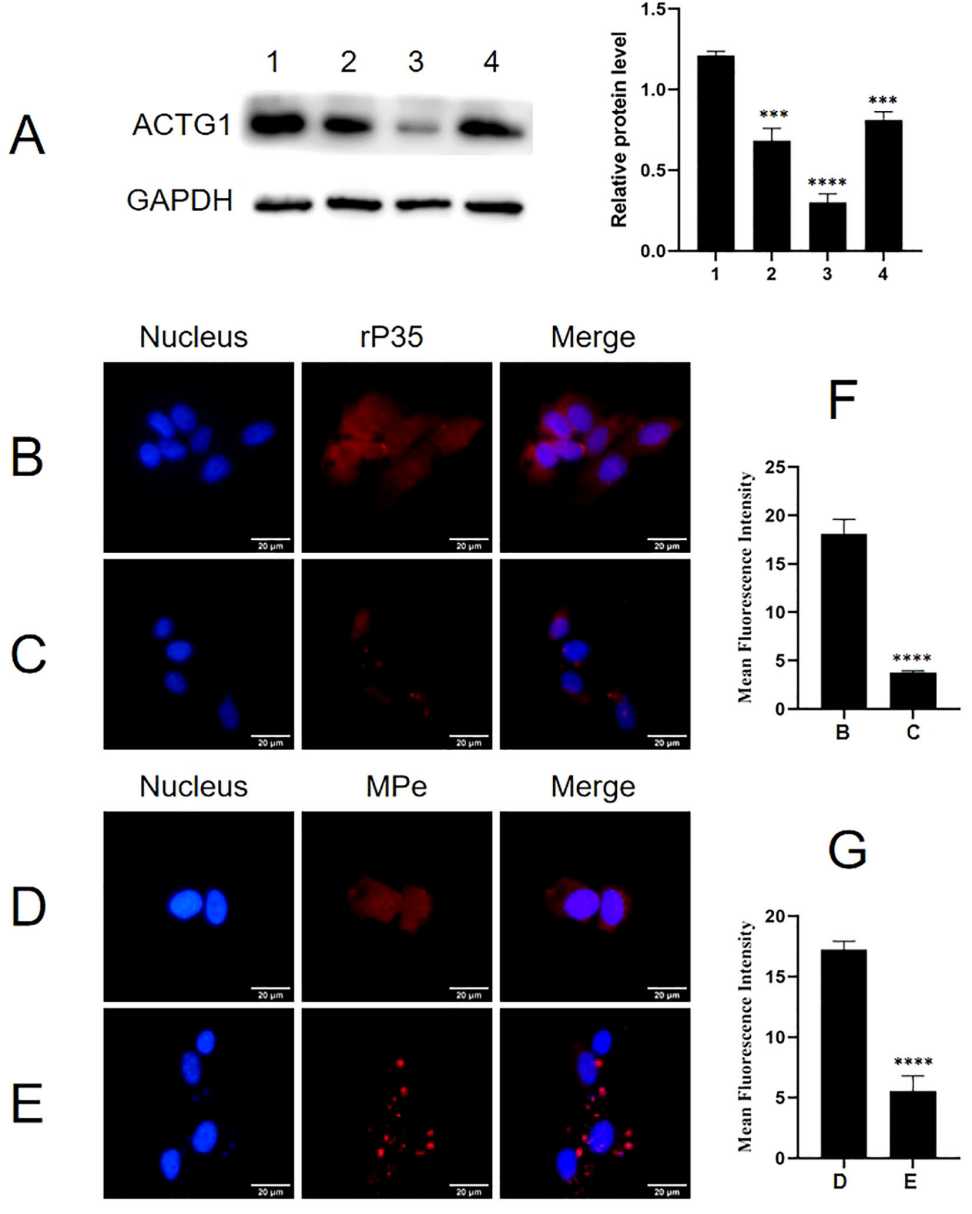
Effect of ACTG1 expression on the adhesion of rP35 and *M. penetrans* to SV-HUC-1 cells. **(A)** Relative protein levels of ACTG1 in SV-HUC-1 cells after transfection with different siRNA duplexes. 1. Control-siRNA; 2. SR300049A; 3. SR300049B; 4. SR300049C. **(B)** Adhesion assay of rP35 on SV-HUC-1 cells treated with Control-siRNA. **(C)** Adhesion assay of rP35 on cells treated with ACTG1-siRNA. **(D)** Adhesion assay of *M. penetrans* on cells treated with Control-siRNA. **(E)** Adhesion assay of *M. penetrans* on cells treated with ACTG1-siRNA. Nuclei were stained with DAPI (blue), and rP35 and *M. penetrans* were stained with Cy3 (red). The images were taken at a magnification of 400× and are representative of multiple areas on multiple slides. **(F, G)** The quantitation of the staining intensities of rP35 **(F)** and *M. penetrans* **(G)** from fluorescence microscopy. ***P < 0.001, ****P < 0.0001.

Adhesion and adhesion inhibition assays were conducted to evaluate whether ACTG1 can inhibit the adhesion of rP35 and *M. penetrans* to SV-HUC-1 cells. As shown in **Figure 6**, both rP35 (**Figure 6A**) and *M. penetrans* (**Figure 6D**) could adhere to the membrane surface of SV-HUC-1 cells. However, the adhesion of rP35 (**Figure 6B**) and *M. penetrans* (**Figure 6E**) to ACTG1-treated cells was decreased. Similarly, the adhesion of rP35 (**Figure 6C**) and *M. penetrans* (**Figure 6F**) to anti-ACTG1 antibody pre-incubated cells were partly inhibited. Quantitative analysis revealed that the average fluorescence intensities for the ACTG1 pre-treatment group and the anti-ACTG1 antibody pre-incubation group were significantly lower than those of the positive control group (P < 0.001; **Figures 6G, H**). Additionally, as shown in **Figure 7A**, the expression of ACTG1 in SV-HUC-1 cells transfected with ACTG1-siRNA was significantly decreased and the adhesion of rP35 (**Figure 7C**) and *M. penetrans* (**Figure 7E**) to cells was also decreased significantly compared with positive controls (**Figures 7B, D**). Quantitative staining intensity analysis confirmed that rP35 and *M. penetrans* exhibited significantly decreased adhesion after ACTG1-siRNA treatment (P < 0.001; **Figures 7F, G**). Collectively, these data indicate that ACTG1 plays a crucial role in mediating the adhesion of rP35 and *M. penetrans* to SV-HUC-1 cells.

### Identification of the functional binding domains of P35

**Figure 8:**
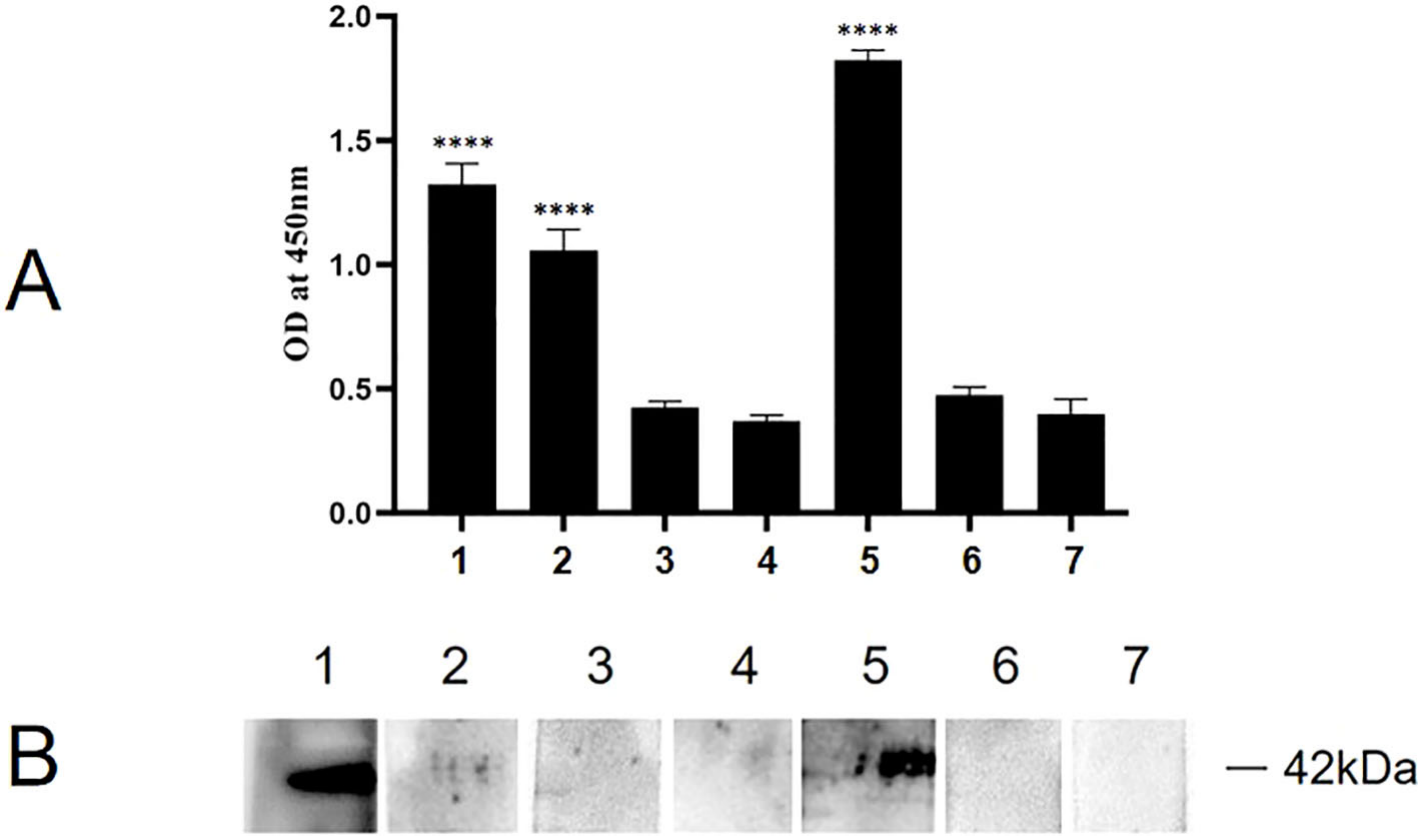
Binding analysis of truncated rMpe-P35 molecules to ACTG1. **(A)** The specific binding of rP35 and synthesized polypeptides to ACTG1 was detected by indirect ELISA. Data points represent the average of experiments performed three times; ****, statistical significance, *p*< 0.0001, compared with the same concentration of control peptide. **(B)** The binding of ACTG1 to rP35 and synthetic peptides were identified by Far-western blot. ACTG1 was isolated by SDS-PAGE and then was transferred to PVDF membranes that were then incubated with full-length rP35 or synthetic peptides, and binding proteins were detected with anti-rP35 antibody or anti-Flag antibody. Lane: 1. Full-length rP35; 2. P35-1; 3. P35-2; 4. P35-3; 5. P35-4; 6. P35-5; 7. control peptide.

Indirect ELISA was carried out to identify the binding functional domains of P35 to ACTG1. As shown in **Figure 8A**, the A_450_ values of P35-1 and P35-4 polypeptide groups were significantly higher than those of the P35-2, P35-3, P35-5 and control peptide group, indicating that P35-1 and P35-4 might bind to ACTG1 *in vitro*. To confirm the results of the ELISA assay, the binding of ACTG1 to rP35 and truncated polypeptides were also detected using far-western blot. As shown in **Figure 8B**, the PVDF membrane incubated with rP35 (Lane 1) and P35-4 (Lane 5) showed obvious bands at 42 kDa, while other polypeptides and control peptide did not have obvious band, which demonstrated that P35-4 peptides can bind to ACTG1.

**Figure 9:**
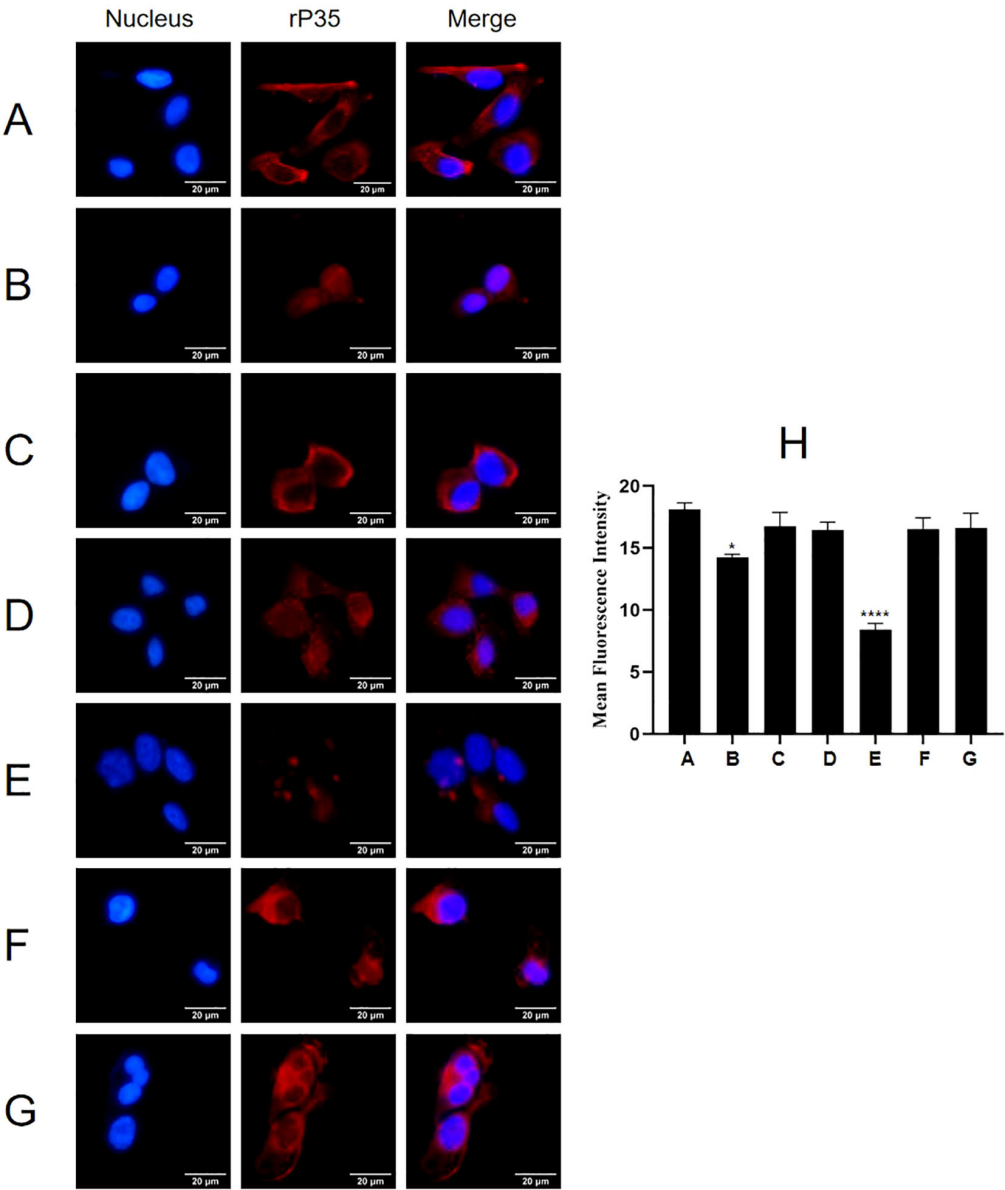
The adhesion of rP35 and 5 synthetic peptides to SV-HUC-1 cells were detected by indirect immunofluorescence assay. **(A)** Adhesion assay of rP35 to SV-HUC-1 cells. **(B)** Peptide rP35-1 treatment group. **(C)** Peptide rP35-2 treatment group. **(D)** Peptide rP35-3 treatment group. **(E)** Peptide rP35-4 treatment group. **(F)** Peptide rP35-5 treatment group. **(G)** Negative peptide treatment group. Nuclei were stained with DAPI (blue), and rP35 was stained with Cy3 (red). The images were taken at a magnification of 40×and are the representative of multiple areas on multiple slides. **(H)** The quantitation of the staining intensities of rP35 from fluorescence microscopy. *P < 0.05, ****P < 0.0001.

Results of adhesion inhibition experiments were shown in **Figure 9**. Compared with the positive control group (**Figure 9A**), the adhesion of rP35 to SV-HUC-1 cells pre-incubated with P35-1 peptide (**Figure 9B**) was relatively reduced, and the adhesion of rP35 to cells pre-incubated with P35-4 (**Figure 9E**) was significantly reduced. After pre-incubated with P35-2 (**Figure 9C**), P35-3 (**Figure 9D**), P35-5 (**Figure 9F**) and control (**Figure 9G**) peptides, the adhesion of rP35 to SV-HUC-1 cells was not significantly reduced compared with the positive control group (**Figure 9A**). Compared with the control group, the mean fluorescence intensity of rP35 attached to cells was statistically significant for P35-1 and P35-4 pre-incubation group (**Figure 9H**). In summary, P35-4 could significantly interact with ACTG1, and peptide P35-1 might interact with ACTG1 with low affinity, while other peptides and control peptide could not bind to ACTG1. These results substantiated that the 35-42 (P35-1) and 179-186 (P35-4) amino acids of P35 lipoprotein were possible binding functional domains that mediate P35 interacting with ACTG1.

## Discussion

This study confirmed that ACTG1 was the main adhesion receptor of P35 lipoprotein on SV-HUC-1 cells and *M. penetrans* interacts with ACTG1 to facilitate adhesion to SV-HUC-1 cells. In addition, we investigated the specific functional binding domain where P35 interacts with ACTG1. The results showed that the amino acid regions 35-42 and 179-186 of the P35 lipoprotein as potenitial binding domains mediating the interaction between P35 and ACTG1, which is the first identification of the functional binding domain of *M. penetrans*. These findings enhance our understanding of the molecular mechanisms underlying *M. penetrans* adhesion and invasion of host cells, providing a foundation for future research aimed at disrupting these interactions to prevent infection.

For many pathogenic *Mycoplasmas*, the initial step in causing disease is the adhesion to host cells through adhesins on polarized organelles (Balish, 2006). The interactions between host cells and *Mycoplasmas* are both complex and diverse. For instance, *M. pneumoniae* relies on P1 and P30 adhesins situated at the tips of their terminal organelles for cellular adhesion (Vizarraga et al., 2020; Seto and Miyata, 2003). *M. mobile*, a fish pathogen, attaches to and invades host cells predominantly through Gli349 adhesins located at the base or neck of their terminal organelles (Kobayashi et al., 2021; Seto and Miyata, 2003). In contrast, *M. penetrans* possesses a distinctive elongated flaker-like tip structure that enables it to adhere to and penetrate the epithelial cells of the genitourinary tract in infected patients (Jurkovic et al., 2013). *M. penetrans* interacts with the host cells by attaching in a highly oriented manner and subsequently internalizing into the cells (Rosengarten et al., 2000). Studies have shown that *M. penetrans* adheres to host cells by LAMP, especially the protein with molecular weight of 35 kDa exposed to the cell surface (Rosengarten et al., 2000; Ferraz et al., 2007). The P35 lipoprotein, the most abundant protein on *M. penetrans* membrane, is distributed uniformly across the plasma membrane rather than being confined to the tip structure (Neyrolles et al., 1999b; Zeiman et al., 2008). which is in stark contrast to P1, P30, P90, and HMW3 of *M. pneumoniae* and *M. genitalium* protein of adhesion (MgPa), as these proteins all cluster at the tip organelles (Liao et al., 2021; Chen et al., 2016). Notably, *M. hominis* lacks a tip structure, and its adhesion protein P50 is distributed throughout the cell surface (Kitzerow et al., 1999). Therefore, the widespread distribution of the P35 lipoprotein on the plasma membrane suggests it may play a crucial role in *M. penetrans* adhesion to host cells. Our results indicated that both rP35 and *M. penetrans* could adhere to SV-HUC-1 cells, and that rabbit anti-rP35 antibodies could partially inhibit this adhesion. These findings suggest that rP35 contains functional domains essential for *M. penetrans* adhesion and interacts with host cells as an adhesion-related protein.

Interactions between mycoplasmal adhesins and host cell receptor proteins are pivotal in understanding the pathogenicity and mechanisms of *Mycoplasmas*. Our previous studies revealed that MgPa interacts with the receptor protein CypA on the membrane of SV-HUC-1 cells, inhibiting apoptosis through the CypA-CD147 pathway and thereby facilitating the early survival of *M. genitalium* within host cell (Liao et al., 2022; Li et al., 2020). Further investigations demonstrated that MgPa suppresses T cell activation via the CypA-Calcineurin-NFAT pathway (Luo et al., 2023). Additionally, we showed that the *M. pneumoniae* adhesin P1 interacts with vimentin on the surface of BEAS-2B cells to mediate adhesion (Peng et al., 2023). These studies collectively indicate that interactions between mycoplasmal adhesins and host receptor proteins are critical for mycoplasma colonization and adhesion. Consequently, this study focused on identifying the receptor and functional binding domains of P35, which possesses both adhesion and antigenic properties, to better understand the mechanisms underlying *M. penetrans* adhesion and invasion of host cells. Results of Modified VOPBA and HPLC-MS showed that ACTG1 and KRT8 may be potential cell receptors for P35. And we confirmed that ACTG1 and KRT8 proteins were localized on the cell membrane and in the cytoplasm of SV-HUC-1 cells. Subsequently, four separate experiments demonstrated that only ACTG1 specifically interacts with P35 and is co-located with *M. penetrans* and rP35 on SV-HUC-1 cells. This suggests that ACTG1 may be a *M. penetrans* P35 lipoprotein receptor on the surface of SV-HUC-1 cells. However, results of Far-Western blot and indirect ELISA showed that rP35 did not interact with KRT8, so we focused only on ACTG1 in subsequent analyses. In this study, there are a few evidences demonstrated that ACTG1 is *M. penetrans* P35 lipoprotein receptor: (I) ACTG1 antibodies can inhibit the ability of rP35 and *M. penetrans* to adhere to and colonize SV-HUC-1 cells; (II) The adhesion of both rP35 and *M. penetrans* to SV-HUC-1 cells decreased after incubation with ACTG1-treated rP35 and *M. penetrans*; (III) The adhesion of rP35 and *M. penetrans* to SV-HUC-1 cells transfected with ACTG1-siRNA was significantly decreased.

Actin is a cytoskeletal protein expressed on the surface of various eukaryotic cells, which participates in a variety of cellular functions including muscle contraction, cell movement, cell adhesion, and cell shape (Pollard and Cooper, 1986; Luo et al., 2014). In vertebrates, there are three main subtypes of actin, namely α, β and γ. Alpha-actin is the major component of the contractile apparatus in muscle tissue. And β-actin and γ- actin commonly coexist in most cell types as mediators of internal cell movement and components of the cytoskeleton (Luo et al., 2014; van Wijk et al., 2003). ACTG1, also known as γ- actin, is one of the six functional actin isomers (Wu et al., 2021). Recent studies have linked ACTG1 to the occurrence, development, and progression of various diseases, including hearing loss and tumors (Miyajima et al., 2020; Richter et al., 2020). In auditory cells, dominant progressive deafness is associated with multiple mutations in ACTG1 (Miyajima et al., 2020). ACTG1 mutations can also cause a brain malformation (Baraitser-Winter syndrome) in humans (Graziani et al., 2023; Kim et al., 2024). Additionally, ACTG1-deficient mice exhibit growth impairments and reduced cell viability (Bunnell and Ervasti, 2010). The abnormal expression of ACTG1 was closely related to the formation, invasion and metastasis of various tumors (Malek et al., 2020; Xiao et al., 2021; Ren et al., 2024). Based on its role in various cancers, ACTG1 is considered a potential biomarker for tumorigenesis and treatment. For example, hepatocellular carcinoma induced by hepatitis B virus X-protein (HBx) was associated with systemic dysregulation of ACTG1 (Sun et al., 2007; Gao et al., 2019). ACTG1 was highly expressed in skin cancer tissues, and it may regulate the proliferation and migration of A431 cells through the ROCK signaling pathway (Dong et al., 2018). In osteosarcoma, abnormal expression of ACTG1 may enhance tumor invasion by affecting microtubule stability, thus negatively affecting patient prognosis (Li et al., 2010). In colorectal cancer cells, ectopic expression of ACTG1 may delay cell adhesion (Liu et al., 2017). These findings suggest that ACTG1 is involved in various cellular processes, including motility and adhesion.

In this study, we found that when the corresponding site of ACTG1 on the surface of host cells is occupied or its expression level is decreased, the amount of *M. penetrans* adhesion to host cells is decreased, which also proves that ACTG1 may participate in cell adhesion. Therefore, occupying the corresponding site of ACTG1 on the surface of host cells in advance or reducing endogenous ACTG1 level will help prevent *M. penetrans* infection. Our study also showed that the 35-42 and 179-186 amino acid regions of P35 lipoprotein are potential binding domains mediating the interaction of P35 and ACTG1. Therefore, how to destroy the interaction binding domain also provides a new treatment option for penetrating *M. penetrans* infection. However, Far-Western blot suggested that P35-1 may not bind specifically to ACTG1, possibly indicating a low-affinity interaction. To resolve this inconsistency, further experiments, such as constructing mutants or performing X-ray crystallography, are necessary to clarify the binding dynamics.

## Data Availability

The original contributions presented in the study are included in the article/**Supplementary Material**. Further inquiries can be directed to the corresponding author.

## Supplementary material

Supplementary Table 1 Identification of rP35-Binding Proteins by HPLC-MS.

Supplementary Figure 1 Expression, purification and identification of recombinant P35 protein. **(A)** SDS-PAGE analysis of rP35 protein expression. Lanes: M. Protein Marker; 1. control, recombinant bacterial lysate, IPTG (-); 2. recombinant bacterial lysate (0.5 mmol/L IPTG); 3. recombinant bacterial lysate supernatants (0.5 mmol/L IPTG); 4. recombinant bacterial lysate precipitation (0.5 mmol/L IPTG); **(B)** SDS-PAGE analysis of rP35 eluent. Lanes: 1. control, effluent liquid; 2-7. the concentrations of imidazole used for elution were 60, 80, 90, 100, 150 mmol/L in sequence. **(C)** SDS-PAGE analysis of purified and concentrated rP35. Lanes: M. Protein Marker; 1. Ultrafiltration enriched rP35 group. **(D)** The recombinant protein P35 was isolated by SDS-PAGE, and electrophoretic transfers were probed by Western blot with anti-His antibody. Lanes: 1. IPTG (-) group; 2. Ultrafiltration enriched rP35 group; 3. empty *E. coli* suspension.

Supplementary Figure 2 Preparation and purification of Anti-rP35 antibody. **(A)** Curve of serum antibody titers in rP35 immunized rabbits. Serum antibody titers of rP35 increased with the number of weeks of immunization, but there was no change in the control group. **(B)** SDS-PAGE analysis of purified anti-rP35 antibodies. **(C)** The recombinant protein P35 was isolated by SDS-PAGE, and electrophoretic transfers were probed by Western blot with purified anti-rP35 antibodies. Lane: 1. control, empty *E. coli* suspension; 2. purified P35 protein group.

## References

[B1] Balish M. F. (2006). Subcellular structures of mycoplasmas. Front. Biosci. 11, 2017–2027. doi: 10.2741/1943 16720287

[B2] Bunnell T. M. Ervasti J. M. (2010). Delayed embryonic development and impaired cell growth and survival in Actg1 null mice. Cytoskeleton (Hoboken) 67, 564–572. doi: 10.1002/cm.20467 20662086 PMC2989386

[B3] Chen Z. Shao X. Dou X. Zhang X. Wang Y. Zhu C.. (2016). Role of the mycoplasma pneumoniae/interleukin-8/neutrophil axis in the pathogenesis of pneumonia. PLoS One 11, e0146377. doi: 10.1371/journal.pone.0146377 26752656 PMC4708980

[B4] Chen Y. Wu Y. Qin L. Yu L. Luo H. Li Y.. (2021). T-B cell epitope peptides induce protective immunity against Mycoplasma pneumoniae respiratory tract infection in BALB/c mice. Immunobiology 226, 152077. doi: 10.1016/j.imbio.2021.152077 33831654

[B5] Deng X. Dai P. Yu M. Chen L. Zhu C. You X.. (2018). Cyclophilin A is the potential receptor of the Mycoplasma genitalium adhesion protein. Int. J. Med. Microbiol. 308, 405–412. doi: 10.1016/j.ijmm.2018.03.001 29551599

[B6] Dong X. Han Y. Sun Z. Xu J. (2018). Actin Gamma 1, a new skin cancer pathogenic gene, identified by the biological feature-based classification. J. Cell Biochem. 119, 1406–1419. doi: 10.1002/jcb.v119.2 28727228

[B7] Ferraz A. S. Belo E. F. Coutinho L. M. Oliveira A. P. De Gaspari E. N. (2007). Rapid and efficient preparation of monoclonal antibodies against 35 kDa lipoprotein of Mycoplasma penetrans. Hybridoma (Larchmt) 26, 92–97. doi: 10.1089/hyb.2006.046 17451357

[B8] Gao B. Li S. Tan Z. Ma L. Liu J. (2019). ACTG1 and TLR3 are biomarkers for alcohol-associated hepatocellular carcinoma. Oncol. Lett. 17, 1714–1722. doi: 10.3892/ol.2018.9757 30675230 PMC6341811

[B9] Gardette M. Touati A. Laurier-Nadalié C. Bébéar C. Pereyre S. (2023). Prevalence of mycoplasma penetrans in urogenital samples from men screened for bacterial sexually transmitted infections. Open Forum Infect. Dis. 10, ofad180. doi: 10.1093/ofid/ofad180 37082616 PMC10111059

[B10] Girón J. A. Lange M. Baseman J. B. (1996). Adherence, fibronectin binding, and induction of cytoskeleton reorganization in cultured human cells by Mycoplasma penetrans. Infect. Immun. 64, 197–208. doi: 10.1128/iai.64.1.197-208.1996 8557340 PMC173746

[B11] Graziani L. Cinnirella G. Ferradini V. Conte C. Bascio F. L. Bengala M.. (2023). A likely pathogenic ACTG1 variant in a child showing partial phenotypic overlap with Baraitser-Winter syndrome. Am. J. Med. Genet. A 191, 1565–1569. doi: 10.1002/ajmg.a.v191.6 36810952

[B12] He J. Wang S. Zeng Y. You X. Ma X. Wu N.. (2014). Binding of CD14 to Mycoplasma genitalium-derived lipid-associated membrane proteins upregulates TNF-α. Inflammation 37, 322–330. doi: 10.1007/s10753-013-9743-7 24068451

[B13] Huang X. Qiao Y. Zhou Y. Ruan Z. Kong Y. Li G.. (2018). Ureaplasma spp. lipid-associated membrane proteins induce human monocyte U937 cell cycle arrest through p53-independent p21 pathway. Int. J. Med. Microbiol. 308, 819–828. doi: 10.1016/j.ijmm.2018.07.005 30033344

[B14] Jurkovic D. A. Hughes M. R. Balish M. F. (2013). Analysis of energy sources for Mycoplasma penetrans gliding motility. FEMS Microbiol. Lett. 338, 39–45. doi: 10.1111/fml.2012.338.issue-1 23066969 PMC3521069

[B15] Kim J. W. Kim S. Y. Lee D. (2024). Ocular findings in Baraitser-Winter syndrome with a *de novo* mutation in the ACTG1 gene: a case report. BMC Ophthalmol. 24, 524. doi: 10.1186/s12886-024-03791-1 39639254 PMC11619142

[B16] Kitzerow A. Hadding U. Henrich B. (1999). Cyto-adherence studies of the adhesin P50 of Mycoplasma hominis. J. Med. Microbiol. 48, 485–493. doi: 10.1099/00222615-48-5-485 10229546

[B17] Kobayashi K. Kodera N. Kasai T. Tahara Y. O. Toyonaga T. Mizutani M.. (2021). Movements of mycoplasma mobile gliding machinery detected by high-speed atomic force microscopy. mBio 12, e0004021. doi: 10.1128/mBio.00040-21 34044587 PMC8262943

[B18] Li Y. Liang Q. Wen Y. Q. Chen L. L. Wang L. T. Liu Y. L.. (2010). Comparative proteomics analysis of human osteosarcomas and benign tumor of bone. Cancer Genet. Cytogenet. 198, 97–106. doi: 10.1016/j.cancergencyto.2010.01.003 20362224

[B19] Li L. Luo D. Liao Y. Peng K. Zeng Y. (2020). Mycoplasma genitalium Protein of Adhesion Induces Inflammatory Cytokines via Cyclophilin A-CD147 Activating the ERK-NF-κB Pathway in Human Urothelial Cells. Front. Immunol. 11, 2052. doi: 10.3389/fimmu.2020.02052 33013867 PMC7509115

[B20] Liao Y. Deng X. Peng K. Dai P. Luo D. Liu P.. (2021). Identification of histone H2B as a potential receptor for Mycoplasma genitalium protein of adhesion. Pathog. Dis. 79(7), ftab053. doi: 10.1093/femspd/ftab053 34755841

[B21] Liao Y. Peng K. Li X. Ye Y. Liu P. Zeng Y. (2022). The adhesion protein of Mycoplasma genitalium inhibits urethral epithelial cell apoptosis through CypA-CD147 activating PI3K/Akt/NF-κB pathway. Appl. Microbiol. Biotechnol. 106, 6657–6669. doi: 10.1007/s00253-022-12146-z 36066653

[B22] Liu Y. Zhang Y. Wu H. Li Y. Zhang Y. Liu M.. (2017). miR-10a suppresses colorectal cancer metastasis by modulating the epithelial-to-mesenchymal transition and anoikis. Cell Death Dis. 8, e2739. doi: 10.1038/cddis.2017.61 28383561 PMC5477594

[B23] Lo S. C. Hayes M. M. Kotani H. Pierce P. F. Wear D. J. Newton P. B. 3rd. (1993). Adhesion onto and invasion into mammalian cells by mycoplasma penetrans: a newly isolated mycoplasma from patients with AIDS. Mod Pathol. 6, 276–280. 8346175

[B24] Lo S. C. Hayes M. M. Tully J. G. Wang R. Y. Kotani H. Pierce P. F.. (1992). Mycoplasma penetrans sp. nov., from the urogenital tract of patients with AIDS. Int. J. Syst. Bacteriol 42, 357–364. doi: 10.1099/00207713-42-3-357 1503969

[B25] Lo S. C. Wang R. Y. Grandinetti T. Zou N. Hayes M. M. Shih J. W.. (2005). Mycoplasma penetrans infections and seroconversion in patients with AIDS: identification of major mycoplasmal antigens targeted by host antibody response. FEMS Immunol. Med. Microbiol. 44, 277–282. doi: 10.1016/j.femsim.2004.12.010 15907449

[B26] Luo Y. Kong F. Wang Z. Chen D. Liu Q. Wang T.. (2014). Loss of ASAP3 destabilizes cytoskeletal protein ACTG1 to suppress cancer cell migration. Mol. Med. Rep. 9, 387–394. doi: 10.3892/mmr.2013.1831 24284654

[B27] Luo D. Luo H. Yan X. Lei A. He J. Liao Y.. (2023). Mycoplasma genitalium Protein of Adhesion Suppresses T Cell Activation via CypA-CaN-NFAT Pathway. Microbiol. Spectr. 11, e0450322. doi: 10.1128/spectrum.04503-22 37074201 PMC10269615

[B28] Malek N. Mrówczyńska E. Michrowska A. Mazurkiewicz E. Pavlyk I. Mazur A. J. (2020). Knockout of ACTB and ACTG1 with CRISPR/Cas9(D10A) technique shows that non-muscle β and γ Actin are not equal in relation to human melanoma cells’ Motility and focal adhesion formation. Int. J. Mol. Sci. 21. doi: 10.3390/ijms21082746 PMC7216121 32326615

[B29] Melgaço A. C. C. Blohem Pessoa W. F. Freire H. P. Evangelista de Almeida M. Santos Barbosa M. Passos Rezende R.. (2018). Potential of maintaining a healthy vaginal environment by two lactobacillus strains isolated from cocoa fermentation. BioMed. Res. Int. 2018, 7571954. doi: 10.1155/2018/7571954 30364031 PMC6186379

[B30] Miyajima H. Moteki H. Day T. Nishio S. Y. Murata T. Ikezono T.. (2020). Novel ACTG1 mutations in patients identified by massively parallel DNA sequencing cause progressive hearing loss. Sci. Rep. 10, 7056. doi: 10.1038/s41598-020-63690-5 32341388 PMC7184572

[B31] Neyrolles O. Chambaud I. Ferris S. Prevost M. C. Sasaki T. Montagnier L.. (1999a). Phase variations of the Mycoplasma penetrans main surface lipoprotein increase antigenic diversity. Infect. Immun. 67, 1569–1578. doi: 10.1128/IAI.67.4.1569-1578.1999 10084988 PMC96498

[B32] Neyrolles O. Eliane J. P. Ferris S. Ayr Florio da Cunha R. Prevost M. C. Bahraoui E.. (1999b). Antigenic characterization and cytolocalization of P35, the major Mycoplasma penetrans antigen. Microbiol. (Reading) 145, 343–355. doi: 10.1099/13500872-145-2-343 10075417

[B33] Peng K. Liao Y. Li X. Zeng D. Ye Y. Chen L.. (2023). Vimentin is an attachment receptor for mycoplasma pneumoniae P1 protein. Microbiol. Spectr. 11, e0448922. doi: 10.1128/spectrum.04489-22 36912679 PMC10100666

[B34] Pollard T. D. Cooper J. A. (1986). Actin and actin-binding proteins. A critical evaluation of mechanisms and functions. Annu. Rev. Biochem. 55, 987–1035. doi: 10.1146/annurev.bi.55.070186.005011 3527055

[B35] Preiswerk B. Imkamp F. Vorburger D. Hömke R. V. Keller P. M. Wagner K. (2020). Mycoplasma penetrans bacteremia in an immunocompromised patient detected by metagenomic sequencing: a case report. BMC Infect. Dis. 20, 7. doi: 10.1186/s12879-019-4723-7 31900105 PMC6942334

[B36] Ren L. Wang L. Yi X. Tan Y. Yi L. He J.. (2024). Ultrasound microbubble-stimulated miR-145-5p inhibits Malignant behaviors of breast cancer cells by targeting ACTG1. Ultrasound Q 40, 136–143. doi: 10.1097/RUQ.0000000000000678 38350033

[B37] Richter C. Mayhew D. Rennhack J. P. So J. Stover E. H. Hwang J. H.. (2020). Genomic amplification and functional dependency of the gamma actin gene ACTG1 in uterine cancer. Int. J. Mol. Sci. 21. doi: 10.3390/ijms21228690 PMC7698702 33217970

[B38] Rosengarten R. Citti C. Glew M. Lischewski A. Droesse M. Much P.. (2000). Host-pathogen interactions in mycoplasma pathogenesis: virulence and survival strategies of minimalist prokaryotes. Int. J. Med. Microbiol. 290, 15–25. doi: 10.1016/S1438-4221(00)80099-5 11043978

[B39] Röske K. Blanchard A. Chambaud I. Citti C. Helbig J. H. Prevost M. C.. (2001). Phase variation among major surface antigens of Mycoplasma penetrans. Infect. Immun. 69, 7642–7651. doi: 10.1128/IAI.69.12.7642-7651.2001 11705944 PMC98858

[B40] Rottem S. (2003). Interaction of mycoplasmas with host cells. Physiol. Rev. 83, 417–432. doi: 10.1152/physrev.00030.2002 12663864

[B41] Sasaki Y. Ishikawa J. Yamashita A. Oshima K. Kenri T. Furuya K.. (2002). The complete genomic sequence of Mycoplasma penetrans, an intracellular bacterial pathogen in humans. Nucleic Acids Res. 30, 5293–5300. doi: 10.1093/nar/gkf667 12466555 PMC137978

[B42] Schwab N. R. Young N. E. Nzenwata D. U. Toh E. Mikulin J. A. Wilson T. J.. (2023). Characterization of virulence-associated traits in mycoplasma penetrans strains acting as likely etiological agents of idiopathic nongonococcal urethritis. J. Infect. Dis. 227, 1050–1058. doi: 10.1093/infdis/jiac505 36588346 PMC10319971

[B43] Seto S. Miyata M. (2003). Attachment organelle formation represented by localization of cytadherence proteins and formation of the electron-dense core in wild-type and mutant strains of Mycoplasma pneumoniae. J. Bacteriol 185, 1082–1091. doi: 10.1128/JB.185.3.1082-1091.2003 12533484 PMC142798

[B44] Srinivasan S. Chambers L. C. Tapia K. A. Hoffman N. G. Munch M. M. Morgan J. L.. (2021). Urethral Microbiota in Men: Association of Haemophilus influenzae and Mycoplasma penetrans With Nongonococcal Urethritis. Clin. Infect. Dis. 73, e1684–e1e93. doi: 10.1093/cid/ciaa1123 32750107 PMC8492123

[B45] Sun Q. Wang Y. Zhang Y. Liu F. Cheng X. Hou N.. (2007). Expression profiling reveals dysregulation of cellular cytoskeletal genes in HBx-induced hepatocarcinogenesis. Cancer Biol. Ther. 6, 668–674. 17873514 10.4161/cbt.6.5.3955

[B46] van Wijk E. Krieger E. Kemperman M. H. De Leenheer E. M. Huygen P. L. Cremers C. W.. (2003). A mutation in the gamma actin 1 (ACTG1) gene causes autosomal dominant hearing loss (DFNA20/26). J. Med. Genet. 40, 879–884. doi: 10.1136/jmg.40.12.879 14684684 PMC1735337

[B47] Vizarraga D. Kawamoto A. Matsumoto U. Illanes R. Pérez-Luque R. Martín J.. (2020). Immunodominant proteins P1 and P40/P90 from human pathogen Mycoplasma pneumoniae. Nat. Commun. 11. doi: 10.1038/s41467-020-18777-y PMC7560827 33057023

[B48] Wang R. Y. Shih J. W. Grandinetti T. Pierce P. F. Hayes M. M. Wear D. J.. (1992). High frequency of antibodies to Mycoplasma penetrans in HIV-infected patients. Lancet 340, 1312–1316. doi: 10.1016/0140-6736(92)92493-Y 1360035

[B49] Wu T. Jia X. Feng H. Wu D. (2021). ACTG1 regulates intervertebral disc degeneration via the NF-κB-p65 and Akt pathways. Biochem. Biophys. Res. Commun. 545, 54–61. doi: 10.1016/j.bbrc.2021.01.057 33545632

[B50] Xiao L. Peng H. Yan M. Chen S. (2021). Silencing ACTG1 expression induces prostate cancer epithelial mesenchymal transition through MAPK/ERK signaling pathway. DNA Cell Biol. 40, 1445–1455. doi: 10.1089/dna.2021.0416 34767732

[B51] Yueyue W. Feichen X. Yixuan X. Lu L. Yiwen C. Xiaoxing Y. (2022). Pathogenicity and virulence of Mycoplasma genitalium: Unraveling Ariadne’s Thread. Virulence 13, 1161–1183. doi: 10.1080/21505594.2022.2095741 35791283 PMC9262362

[B52] Zeiman E. Tarshis M. Rottem S. (2008). Mycoplasma penetrans under nutritional stress: influence on lipid and lipoprotein profiles and on the binding to and invasion of HeLa cells. FEMS Microbiol. Lett. 287, 243–249. doi: 10.1111/j.1574-6968.2008.01322.x 18754786

[B53] Zeng Y. Liu L. He J. Liu Y. Zhu C. You X.. (2012). Screening and identification of the mimic epitope of the adhesion protein of Mycoplasma genitalium. Can. J. Microbiol. 58, 898–908. doi: 10.1139/w2012-057 22716192

