# Supplementary material for: Screening the receptors for Mycoplasma penetrans P35 lipoprotein and characterization of its functional binding domains
Source: Front Cell Infect Microbiol. 2025 Mar 17;15:1525789. doi: 10.3389/fcimb.2025.1525789 (PMC11955645; doi:10.3389/fcimb.2025.1525789)
Supplement: Supplementary Table 1 — Identification of rP35-Binding Proteins by HPLC-MS. [file Table1.docx]

Supplementary Table 1. Identification of rP35-Binding Proteins by HPLC-MS

|  | Accession | Score | Mass | Matches | Sequences | emPAI | Protein description |
| --- | --- | --- | --- | --- | --- | --- | --- |
| 1 | ACTG_HUMAN | 10778 | 42108 | 462 | 31 | 289.69 | Actin, cytoplasmic 2  OS=Homo sapiens OX=9606 GN=ACTG1 PE=1 SV=1 |
| 2 | K2C8_HUMAN | 6308 | 53671 | 281 | 34 | 52.86 | Keratin, type II cytoskeletal 8  OS=Homo sapiens OX=9606 GN=KRT8 PE=1 SV=7 |
