# Supplementary figures and images for: Screening the receptors for Mycoplasma penetrans P35 lipoprotein and characterization of its functional binding domains
Source: Front Cell Infect Microbiol. 2025 Mar 17;15:1525789. doi: 10.3389/fcimb.2025.1525789 (PMC11955645; doi:10.3389/fcimb.2025.1525789)

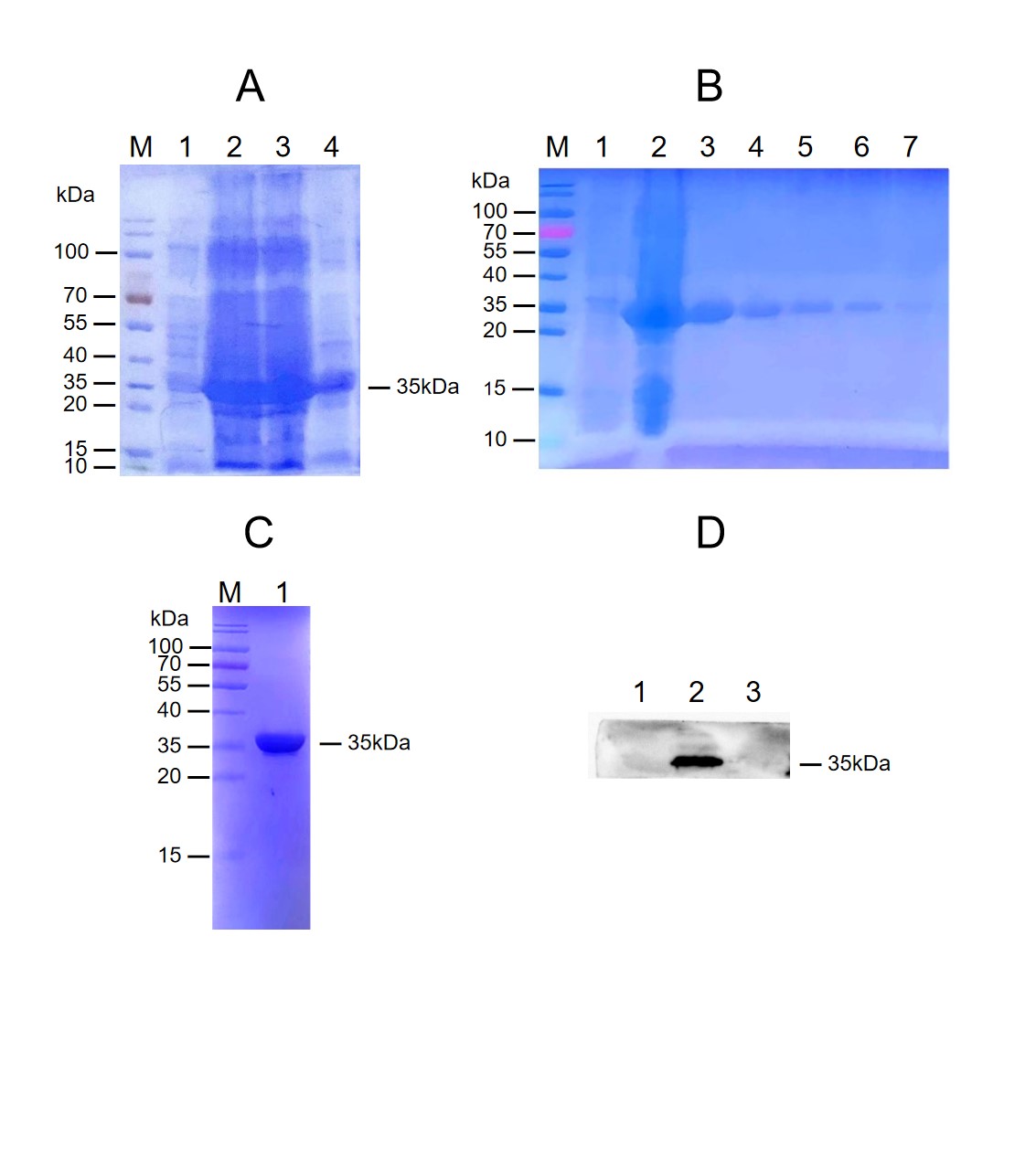

Supplement: Supplementary Figure 1 — Expression, purification and identification of recombinant P35 protein. (A) SDS-PAGE analysis of rP35 protein expression. Lanes: M. Protein Marker; 1. control, recombinant bacterial lysate, IPTG (-); 2. recombinant bacterial lysate (0.5 mmol/L IPTG); 3. recombinant bacterial lysate supernatants (0.5 mmol/L IPTG); 4. recombinant bacterial lysate precipitation (0.5 mmol/L IPTG); (B) SDS-PAGE analysis of rP35 eluent. Lanes: 1. control, effluent liquid; 2-7. the concentrations of imidazole used for elution were 60, 80, 90, 100, 150 mmol/L in sequence. (C) SDS-PAGE analysis of purified and concentrated rP35. Lanes: M. Protein Marker; 1. Ultrafiltration enriched rP35 group. (D) The recombinant protein P35 was isolated by SDS-PAGE, and electrophoretic transfers were probed by Western blot with anti-His antibody. Lanes: 1. IPTG (-) group; 2. Ultrafiltration enriched rP35 group; 3. empty E. coli suspension. [file Image1.jpeg]

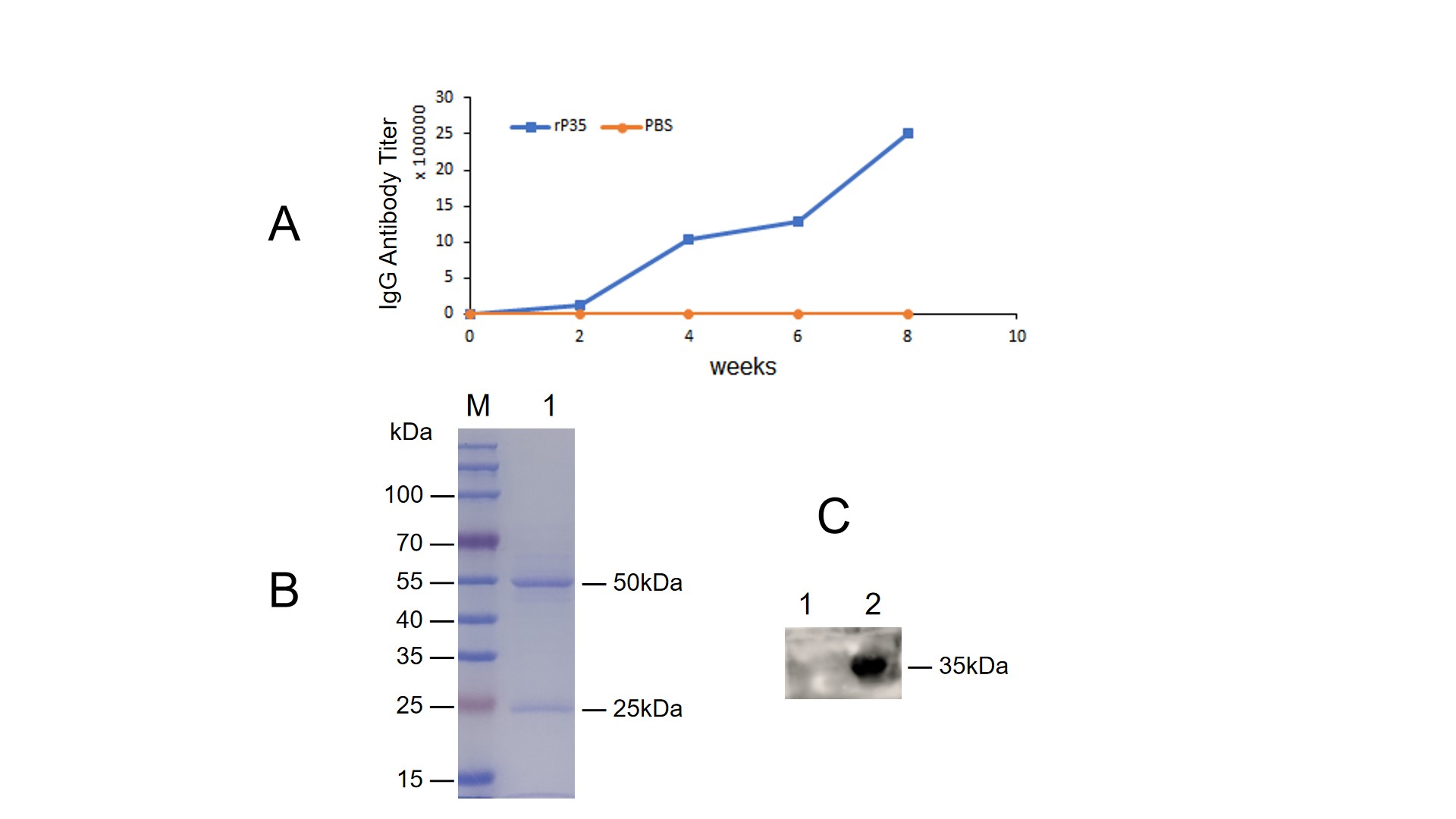

Supplement: Supplementary Figure 2 — Preparation and purification of Anti-rP35 antibody. (A) Curve of serum antibody titers in rP35 immunized rabbits. Serum antibody titers of rP35 increased with the number of weeks of immunization, but there was no change in the control group. (B) SDS-PAGE analysis of purified anti-rP35 antibodies. (C) The recombinant protein P35 was isolated by SDS-PAGE, and electrophoretic transfers were probed by Western blot with purified anti-rP35 antibodies. Lane: 1. control, empty E. coli suspension; 2. purified P35 protein group. [file Image2.jpeg]
